# Molecular portraits of patients with intrahepatic cholangiocarcinoma who diverge as rapid progressors or long survivors on chemotherapy

**DOI:** 10.1136/gutjnl-2023-330748

**Published:** 2023-09-27

**Authors:** Colm J O'Rourke, Massimiliano Salati, Colin Rae, Guido Carpino, Holly Leslie, Antonio Pea, Maria G Prete, Luca R Bonetti, Francesco Amato, Robert Montal, Rosie Upstill-Goddard, Colin Nixon, Paula Sanchon-Sanchez, Paolo Kunderfranco, Daniela Sia, Eugenio Gaudio, Diletta Overi, Stefano Cascinu, Dan Hogdall, Sian Pugh, Enric Domingo, John N Primrose, John Bridgewater, Andrea Spallanzani, Fabio Gelsomino, Josep M Llovet, Diego F Calvisi, Luke Boulter, Francesco Caputo, Ana Lleo, Nigel B Jamieson, Gabriele Luppi, Massimo Dominici, Jesper B Andersen, Chiara Braconi

**Affiliations:** 1 Biotech Research and Innovation Centre (BRIC), University of Copenhagen, Department of Health and Medical Sciences, Copenhagen, Denmark; 2 Division of Oncology, Department of Oncology and Hematology, University Hospital Modena, Modena, Italy; 3 Clinical and Experimental Medicine, University of Modena and Reggio Emilia, Modena, Italy; 4 School of Cancer Sciences, University of Glasgow, Glasgow, UK; 5 Department of Anatomical, Histological, Forensic Medicine and Orthopaedic Sciences, Sapienza University of Rome, Roma, Italy; 6 Division of Pathology, University of Modena and Reggio Emilia, Modena, Italy; 7 Cancer Biomarkers Research Group, Department of Medical Oncology, Hospital Universitari Arnau de Vilanova, Lleida, Spain; 8 Cancer Research UK Beatson Cancer Research Institute, Glasgow, UK; 9 Bioinformatics Unit, IRCCS Humanitas Research Hospital, Rozzano, Italy; 10 Liver Cancer Translational Research Laboratory, BCLC Group, Liver Unit and Pathology Department, Icahn School of Medicine at Mount Sinai, New York, New York, USA; 11 Medical Oncology, IRCCS Humanitas Research Hospital, Milan, Italy; 12 Department of Oncology, Herlev Hospital, Herlev, Denmark; 13 Addenbrooke's Hospital, Cambridge, UK; 14 Department of Oncology, University of Oxford, Oxford, UK; 15 Surgery, University of Southampton, Southampton, UK; 16 Department of Oncology, University College London, London, UK; 17 Translational Research in Hepatic Oncology, Liver Unit, IDIBAPS, Hospital Clínic, University of Barcelona, Barcelona, Spain; 18 Icahn School of Medicine at Mount Sinai, New York, New York, USA; 19 Institució Catalana de Recerca i Estudis Avançats (ICREA), Barcelona, Spain; 20 Institute of Pathology, University of Regensburg Faculty of Medicine, Regensburg, Germany; 21 Medical, Surgical, and Clinical Sciences, University of Sassari, Sassari, Italy; 22 MRC HGU, The University of Edinburgh MRC Institute of Genetics and Molecular Medicine, Edinburgh, UK; 23 CRUK Scotland Cancer Centre, Glasgow-Edinburgh, UK; 24 Department of Biomedical Sciences, Humanitas University, Milan, Italy; 25 Internal Medicine and Hepatology Unit, Department of Gastroenterology, IRCCS Humanitas Research Hospital, Milan, Italy; 26 Beatson West of Scotland Cancer Centre, Glasgow, UK

**Keywords:** chemotherapy, liver, cholangiocarcinoma

## Abstract

**Objective:**

Cytotoxic agents are the cornerstone of treatment for patients with advanced intrahepatic cholangiocarcinoma (iCCA), despite heterogeneous benefit. We hypothesised that the pretreatment molecular profiles of diagnostic biopsies can predict patient benefit from chemotherapy and define molecular bases of innate chemoresistance.

**Design:**

We identified a cohort of advanced iCCA patients with comparable baseline characteristics who diverged as extreme outliers on chemotherapy (survival <6 m in rapid progressors, RP; survival >23 m in long survivors, LS). Diagnostic biopsies were characterised by digital pathology, then subjected to whole-transcriptome profiling of bulk and geospatially macrodissected tissue regions. Spatial transcriptomics of tumour-infiltrating myeloid cells was performed using targeted digital spatial profiling (GeoMx). Transcriptome signatures were evaluated in multiple cohorts of resected cancers. Signatures were also characterised using in vitro cell lines, in vivo mouse models and single cell RNA-sequencing data.

**Results:**

Pretreatment transcriptome profiles differentiated patients who would become RPs or LSs on chemotherapy. Biologically, this signature originated from altered tumour-myeloid dynamics, implicating tumour-induced immune tolerogenicity with poor response to chemotherapy. The central role of the liver microenviroment was confrmed by the association of the RPLS transcriptome signature with clinical outcome in iCCA but not extrahepatic CCA, and in liver metastasis from colorectal cancer, but not in the matched primary bowel tumours.

**Conclusions:**

The RPLS signature could be a novel metric of chemotherapy outcome in iCCA. Further development and validation of this transcriptomic signature is warranted to develop precision chemotherapy strategies in these settings.

**WHAT IS ALREADY KNOWN ON THIS TOPIC**

- Cholangiocarcinoma (CCA) patient management continues to be dominated by an all-comer approach to chemotherapy in first-line despite heterogeneous benefit. Our inability to quantify the chemosensitivity of patients’ disease remains a bottleneck to optimising their clinical management. Increasing knowledge of the molecular bases behind chemosensitivity can aid development of novel therapeutic strategies.

**WHAT THIS STUDY ADDS**

- Pretreatment transcriptomic profiles of diagnostic biopsies differentiate intrahepatic CCA patients who become rapid progressors or long survivors on chemotherapy. The RPLS signature is associated with benefit from cytotoxic agents for patients with primary and liver-metastatic tumours, indicating a precision chemotherapy strategy may be feasible and identifying candidate therapeutic targets to boost chemosensitivity.

**HOW THIS STUDY MIGHT AFFECT RESEARCH, PRACTICE OR POLICY**

- Pending further validation, the RPLS signature could provide a clinical-grade tool to inform on chemotherapy benefit before starting treatment, foregoing unnecessary toxicities from a regimen of limited therapeutic benefit on a patient-by-patient basis. In addition, it unveils the biology behind different long-term outcomes in patients receiving chemotherapy, providing the bases for development of novel therapeutics.

## Introduction

Intrahepatic cholangiocarcinoma (iCCA) is a family of rare, heterogeneous tumours arising from the intrahepatic biliary tree. Incidence and mortality rates of iCCA appear to be increasing.^1 2^ Due to asymptomatic development, typical presentation without known risk factors and lack of early diagnostic biomarkers, more than 50% of patients are diagnosed with locally advanced and metastatic disease.^3^ In this setting, systemic chemotherapy with gemcitabine and cisplatin remains the standard-of-care in first-line,^4^ with reported additive benefit of durvalumab and pembrolizumab for a niche of patients.^5 6^ Overall benefit from FOLFOX in second-line^7^ and capecitabine in the adjuvant setting^8^ reinforce chemotherapy as central to patients’ management throughout their disease trajectory. However, benefit from cytotoxic agents is heterogeneous. In the ABC-02 trial, 11% of biliary tract cancer (BTC) patients were alive at 2 years following enrolment, greater than double the median overall survival (OS).^4 9^ These long survivors (LSs) are contrasted with data indicating that chemotherapy fails to achieve disease control in 25%–28% of advanced BTC patients.^4 10^ Understanding and predicting benefit of chemotherapy remains a critical unmet need for patients with iCCA, especially as novel targeted therapies become available in second-line for specific tumour genotypes^11 12^ and might be superior in first-line for patients unlikely to benefit from chemotherapy.^13^

Precision approaches improve the outcome of patients with iCCA,^14^ but are currently limited to targeted therapies. With an all-comer approach for first-line chemotherapy, it remains unclear which patients will not benefit from this regimen, with implications for optimal treatment and quality of life. Potentially actionable genomic alterations occur in up to 52% of iCCA,^15^ but corresponding therapies are only available following progression on chemotherapy, a rate-limiting step as many patients deteriorate as a result of disease progression. Unlike targeted therapies with clear DNA-based indications in single genes, the molecular basis of innate sensitivity to chemotherapy remains unclear. DNA profiling of tumours is restricted to detecting genetic alterations in tumour cells, omitting the important impact of non-genetic tumour alterations and non-genetic microenvironment alterations on treatment outcome.^16^ Transcriptome profiling captures a more holistic overview of the tumour biology (cell composition and behaviour) and is gaining clinical traction due to successes in matching patients to novel therapies and predicting their outcomes.^17^

We have integrated transcriptomic profiling of bulk and geospatially macrodissected diagnostic biopsies with digital pathology and digital spatial profiling in a cohort of clinically matched iCCA patients with extreme divergent outcomes on chemotherapy. This led to identification of the RPLS gene expression signature as a candidate metric of poor clinical outcome and innate chemoresistance. Modelling the RPLS signature in cell lines, single cell RNA-sequencing data, animal models and bulk transcriptomic data from iCCA implicated tumour-induced immune tolerogenicity as a defining hallmark of rapid progression on chemotherapy, as well as establishing a robust and feasible tool to validate for the development of precision chemotherapy in this rare cancer type.

## Materials and methods

Comprehensive information on patients and methods are provided online in online supplemental information.

10.1136/gutjnl-2023-330748.supp1 Supplementary data

## Results

### The RPLS cohort

**Figure 1:**
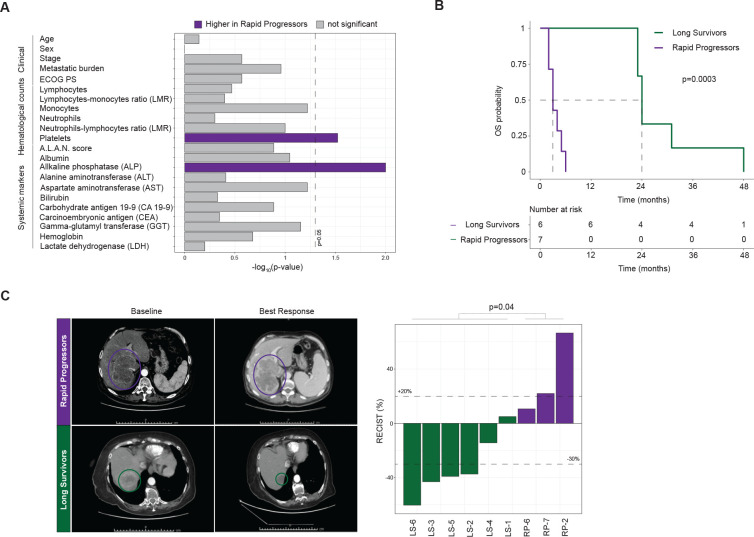
Clinical characteristics and chemotherapy response of patients with intrahepatic cholangiocarcinoma in the RPLS cohort. (A) Kaplan-Meier survival curves with log-rank statistics for overall survivalin the RPLS cohort. (B) Barplot of statistical differences in baseline characteristics between rapid progressor (RP) and long survivor (LS) patients. (C) Representative baseline and best response CT images for an RP and an LS patient. Barplot of best radiological response (Response Evaluation Criteria in Solid Tumours (RECIST)1; Welch t-test). Disease was not measurable for one RP patient, while three RP patients had clinical progression without radiological confirmation (RP-1/RP-3/RP-4/RP-5). ALAN, actual neutrophil count; lymphocyte-monocyetesratio; neutrophil-lymphocytesratio; albumin.

Survival on chemotherapy is heterogeneous among patients with iCCA, including those with comparable clinical features at diagnosis.^9 18^ To identify pretreatment molecular features associated with chemotherapeutic outcome that are currently overlooked in the clinic, we identified a cohort of patients with advanced iCCA who diverged as rapid progressors (RP; n=7) or LS (LS; n=6) on chemotherapy. All RP patients survived less than 6 months (half the median OS reported in the ABC-02 trial^4^), whereas all LS patients survived more than 23 months (double the median OS reported in the ABC-02 trial) (p=0.0003; figure 1A). Critically, these patients did not differ in baseline clinical features established during diagnostic workup (figure 1B, online supplemental table S1). No differences were found in haematological or systemic biochemical features, with the exception of higher platelets (p=0.03) and alkaline phosphatase (ALP) levels (p=0.01) in RP patients. All patients were treated with platinum-combination chemotherapy in first line, leading to greater radiological responses in LS patients (p=0.04; figure 1C). Of note, the response rate in the LS cohort was 66%, more than double compared with an unselected population, suggesting that the differences in the long-term control of the disease are not only related to slow growing tumours. Overall, LS patients received a greater number of lines of chemotherapy (p=0.002; online supplemental figure S1A,B). Collectively, the RPLS cohort epitomises extreme divergent outcomes on chemotherapy in an otherwise homogeneous patient population. As such, despite being of small size, we hypothesised that this cohort provides a prime setting, in which to apply molecular profiling to understand benefit from standard-of-care chemotherapy, as well as potentially overall prognosis.

10.1136/gutjnl-2023-330748.supp2 Supplementary data

10.1136/gutjnl-2023-330748.supp3 Supplementary data

### Transcriptomic profiles of pretreatment biopsies differentiate RPs and LSs

**Figure 2:**
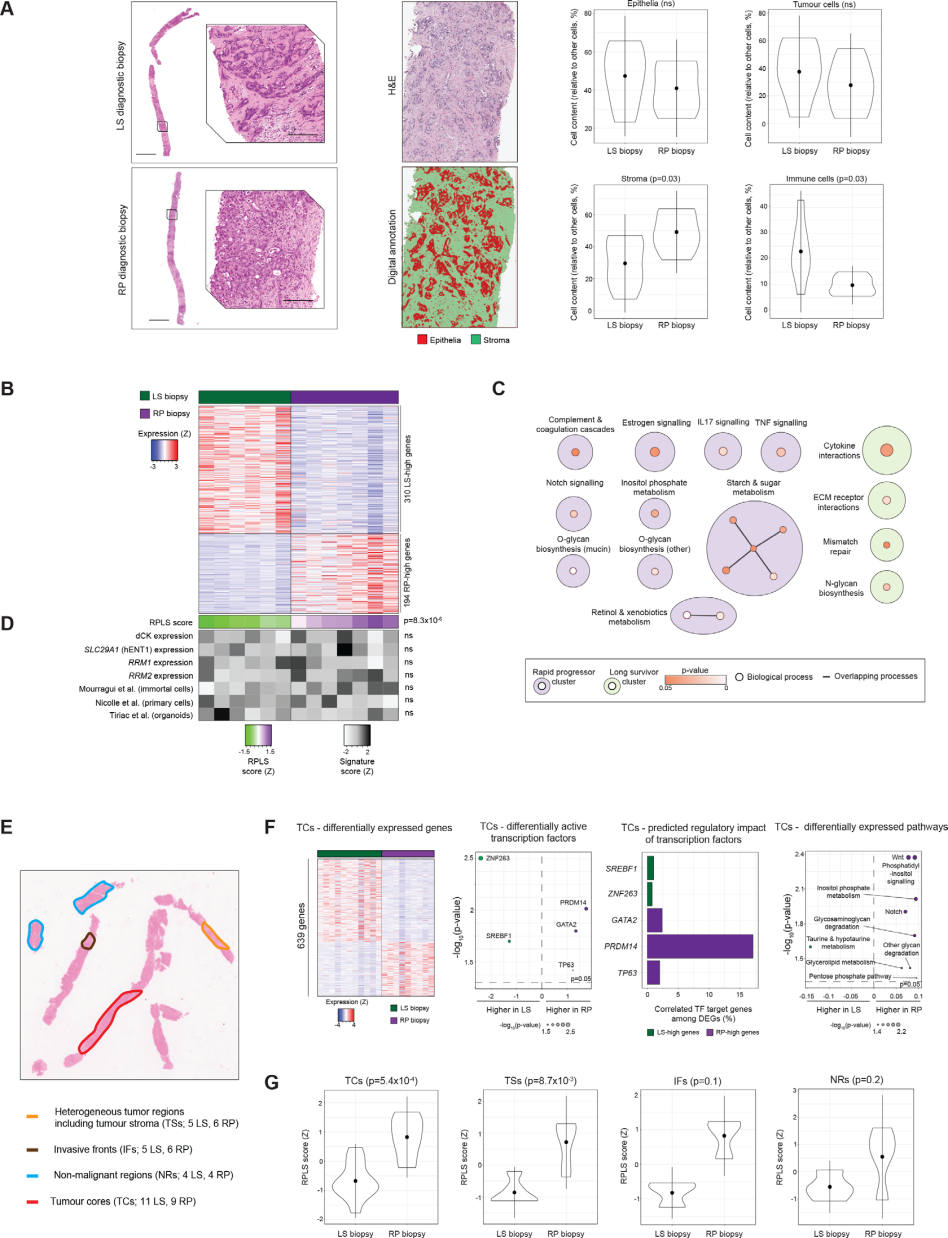
Histopathological and transcriptomic profiles of diagnostic biopsies from the RPLS cohort. (A) Representative H&E images (scale bars: 2 mm—top; 200 µm—bottom), characterisation of the epithelial component of diagnostic biopsies in each region of interest, and differential composition in tumourous, stromal and immune cells following cell segmentation (LS: n=6; RP: n=7; Welch t-test). (B) Heatmap of 504 differentially expressed genes (≥2 fold change, p<0.05; Wilcoxon) between LS and RP biopsies. (C) KEGG pathway over-representation analysis of LS-high and RP-high genes using EnrichmentMap. Overlapping pathways are connected by lines and annotated under a common theme using AutoAnnotate. KEGG: Kyoto Encyclopaedia of Genes and Genomes. (D) Heatmap and differential expression analysis of the RPLS score and previously published metrics of gemcitabine sensitivity in the RPLS cohort. P values were derived by Wilcoxon test. (E) Representative H&E stain of a diagnostic biopsy, indicating histological regions targeted by macrodissection. (F) Differentially expressed genes (≥2 fold change, p<0.05; Wilcoxon), differentially active transcription factors (p<0.05, Wilcoxon test; DoRothEA), differentially expressed pathways (p<0.05, Wilcoxon test; ssGSEA of KEGG and Hallmarks gene lists), and differentially active cytokines (p<0.05, Wilcoxon test; CytoSig) between RP and LS tumour cores (TCs; 11 LS, 9 RP). (G) Differential expression of the bulk tissue RPLS signature in TCs, tumour stroma (TSs; 5 LS, 6 RP), invasive fronts (IFs; 3 LS, 3 RP) and non-malignant regions (NRs; 4 LS, 4 RP) from RP and LS biopsies (Wilcoxon test). LS, long survivor; RP, rapid progressor.

We retrieved the pretreatment, diagnostic liver biopsies for patients in the RPLS cohort and performed digital histopathological evaluation (figure 2A). Pixel classification of the entire biopsy tissues showed no differences between RP and LS biopsies in tumorous (p=0.90), epithelial (p=0.39) or stromal (p=0.39) content. Cell detection and classification analysis also revealed no differences in the total number of epithelial (p=0.43) or tumour cells (p=0.39). However, RP biopsies had higher stromal (p=0.03) and lower immune cell (p=0.03) content, suggesting an association between microenvironment composition and chemotherapy outcome, consistently with previous studies.^19 20^

Next, we performed whole-transcriptome profiling of the bulk biopsies using Tempo-seq, a sequencing technology compatible with the limited and fragmented RNA retrievable from archival FFPE biopsies. In total, 504 genes were differentially expressed between RP and LS biopsies (fold-change≥2, p<0.05), including 310 genes higher expressed in LS (‘LS-high’) and 194 genes higher expressed in RP (‘RP-high’) tissues with distinct biological functions (figure 2B,C, online supplemental table S2). Expression of RP-high and LS-high genes anticorrelated in the biopsies (Spearman’s r=−0.92, p=9.4×10^−6^), suggesting opposing biological functions that are associated with chemotherapy outcome (online supplemental figure S2A). Therefore, we derived a formula using the RPLS signature genes ([log_2_(ΣRP-high genes)−log_2_(ΣLS-high genes)]_z-score_), with resulting RPLS scores being higher in RP compared with LS biopsies (p=8.3×10^−6^; figure 2D). As such, we hypothesised that the RPLS signature might represent a metric of the innate chemoresistance potential of iCCA. Inclusion of pretreatment systemic features that differed between patient subgroups (platelets, ALP; figure 2B) or an optimised systemic signature (defined by AIC backwards elimination using all haematological and systemic features; figure 2C) did not improve the predictive performance of the RPLS score in multivariable analysis (online supplemental figure S2B). This further supports the utility of the RPLS score during diagnostic workup of patients with advanced iCCA. Expression of genes involved in gemcitabine uptake/metabolism and previous transcriptomic predictors of chemosensitivity in other cancers failed to differentiate RP and LS biopsies (figure 2D). Collectively, these observations argue that the pretreatment molecular features associated with chemotherapy outcome are distinct in iCCA compared with other cancers, implicating disease and/or context (liver) specificity with treatment response.

10.1136/gutjnl-2023-330748.supp4 Supplementary data

### Geospatially distinct biopsy regions harbour unique transcriptional programmes that differentiate RP and LS patients

Bulk biopsy transcriptomes capture global signalling but lose biological resolution of specific histopathological regions within the tissues. To address this limitation, we performed geospatial macrodissection followed by whole-transcriptome profiling (Tempo-seq) of tumour cores (TCs; 11 LS, 9 RP), tumour stroma (TSs; 5 LS, 6 RP), invasive fronts (IFs; 3 LS, 3 RP) and non-malignant regions (NRs; 4 LS, 4 RP) from the same RPLS biopsies (figure 2E, online supplemental figure S3). Intrasample transcriptomic heterogeneity and phylotranscriptomic analyses did not differentiate RP and LS biopsies, indicating that intrasample heterogeneity was not associated with outcomes (online supplemental figure S4A,B).

10.1136/gutjnl-2023-330748.supp5 Supplementary data

10.1136/gutjnl-2023-330748.supp6 Supplementary data

Intertumour heterogeneity is dictated by differential gene and pathway expression, modulated by cell-intrinsic transcriptional programmes. Therefore, we identified differentially expressed genes and pathways for each of the macrodissected regions, as well as predicting transcription factor (TF) activities. No significant differences were found for IFs, so these samples are excluded from further discussion.

In TCs, 639 genes (388 LS-high, 251 RP-high) were differentially expressed between LS and RP biopsies (figure 2F). TF activities of PRDM14, GATA2 and TP63 were higher in RP tissues, potentially regulating 17%, 2% and 2% of the RP-high genes, respectively. In LS biopsies, there was increased activity of SREBF1 and ZNF263, each potentially controlling expression of 1% of the LS-high genes. Within these tumour cell-enriched regions, Notch and Wnt pathways were elevated in RP patients, both developmental programmes associated with poor prognosis in iCCA with incremental potential for druggability.^21 22^

In TSs, LS and RP biopsies differed in expression of 704 genes (637 LS-high, 67 RP-high) (online supplemental figure S5A). CDX2 and KLF4 were more active in RP TSs, potentially regulating 9% and 1.5% of the RP-high genes, respectively. ZNF263 was more active in LS TSs and may control expression of approximately 4% of the LS-high genes. Notch signalling was higher in RP TSs, whereas metabolic processes and MYC targets were higher expressed in LS TS regions. Notably, previously reported signatures of cancer-associated fibroblasts (CAFs) in iCCA^23^ did not differ between TSs in our cohort (online supplemental figure S5B).

10.1136/gutjnl-2023-330748.supp7 Supplementary data

In NRs, the expression of 269 genes (251 LS-high, 45 RP-high) differed between LS and RP biopsies (online supplemental figure S5C). Activities of CEBPB and E2F4 were elevated in RP NRs, potentially regulating 4% and 20% of the RP-high genes, respectively. PAX6 was more active in LS NRs and may control expression of 6% of LS-high genes. Diverse processes were more highly expressed in RP (fructose and mannose metabolism, G2M checkpoint, MTORC1 signalling) and LS (cytokine receptor interactions, haematopoietic cell lineage) NRs, the latter suggesting more widespread immune activity in the liver of LS patients.

Approximately one in four biopsies fail molecular profiling in BTCs due to low tumour cellularity.^24^ Evaluation of the RPLS signature (derived from bulk biopsy) in macrodissected biopsy regions revealed RPLS scores to be elevated in both TCs (p=5.4×10^−4^) and TSs (p=8.7×10^−3^) from RP patients, but not in IFs (p=0.1) or NRs (p=0.2) (figure 2G). These data suggest that the RPLS signature is tumour-specific and may be sufficiently assessed in stroma-rich biopsies containing relatively few tumour cells, a critical limitation of DNA-based biopsy profiling in the clinic today.

### The RPLS signature originates from tumour-intrinsic programmes associated with innate immune dysfunction

**Figure 3:**
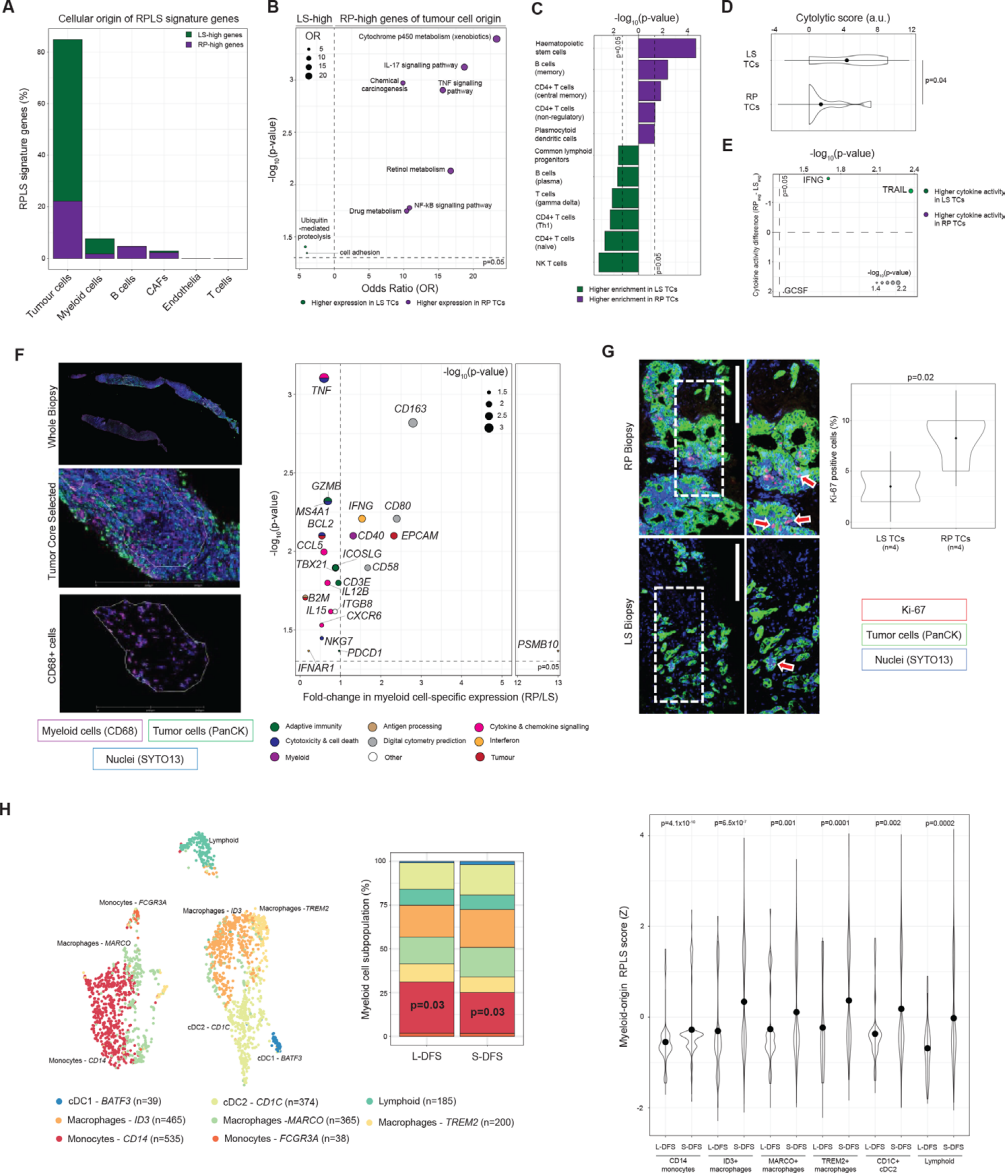
Cellular origins and tumour-immune dynamics associated with the RPLS signature. (A) Barplot of cell type-associations of RPLS signature genes. Genes were only assigned to cell types if their associations were supported by two independent single cell RNA-sequencing datasets. (B) KEGG pathway over-representation analysis of tumour-origin RPLS signature genes. (C) Differential enrichment (Wilcoxon test) of immune cell type signatures in rapid progressor (RP) and long survivor (LS) tumour cores (TCs) determined by cellular deconvolution (xCell). (D) Differential cytolytic scores (Wilcoxon test) between RP and LS TCs. (E) Differential cytokine activities (Wilcoxon test) between RP and LS TCs determined by CytoSig. (F) Representative multiplex immunofluorescence images of TCs undergoing RNA extraction from myeloid (CD68+) myeloid cells using the Digital Spatial Profiling (GeoMx) platform with regions of interest identifed. Volcano plot of differentially expressed genes (Immune Pathways Panel (NanoString) plus 5 custom targets derived from digital cytometry) in tumour-infiltrating myeloid cells from LS (n=6) and RP (n=6) TCs. P values were computed by Wilcoxon test. (G) Representative Ki-67 staining in an LS and RP TCs, including differential proliferation analysis (Welch t-test). (H) tSNE plot of myeloid subpopulations identified in immune-enriched single cell RNA-sequencing data from three resected iCCA. Frequency barplot (p values from χ^2^ test) comparing the abundance of myeloid subpopulations in patients without (long disease-free survival, L-DFS) and with (short disease-free survival, S-DFS) recurrence under adjuvant treatment with capecitabine. Differential expression (Wilcoxon test) of the myeloid-origin RPLS signature in myeloid subpopulations. iCCA, intrahepatic cholangiocarcinoma.

The RPLS signature can originate from differences in cell composition and/or behaviour. To investigate this, we pursued a digital cytometry approach (CIBERSORTx) to infer the cellular origin(s) of RPLS signature genes in our biopsies. Among assignable genes, the RPLS signature predominantly originated from tumour cells, followed by tumour-associated myeloid cells, B cells and CAFs (figure 3A). RP-high genes originating from tumour cells were over-represented in immune signalling pathways (IL-17, NFkB, TNF) and drug metabolism (figure 3B), highlighting two plausible mechanisms (impaired immunogenic cell death, enhanced metabolic inactivation) undermining chemotherapy efficacy.^16 25^

As these immune pathways typically require heterotypic signalling between tumour and immune cells, we hypothesised that defective tumour-innate immune dynamics are a defining characteristic of RP tumours. Consistent with this, immune infiltrates significantly differed between LS and RP TCs (figure 3C). Cytolytic scores were higher in LS TCs (p=0.04), indicating proficient anti-tumour cytotoxicity in these biopsies (figure 3D). Cytokine activity profiles also differed between LS and RP TCs (figure 3E). The interferon responsible for activation of antitumour immunity, interferon-γ (IFNG; p=0.02), and the proapoptotic cytokine, TNF-related apoptosis-inducing ligand (TRAIL; p=0.004), were both more active in LS TCs, indicating effective cytotoxicity and cell death. In contrast, granulocyte colony-stimulating factor (GSCF), a promyelopoiesis and anti-inflammatory cytokine associated with myeloid-derived suppressor cells,^26^ had increased activity in RP TCs (p<0.05), supporting an immunosuppressive phenotype in these tumours.

To experimentally verify immune cell dysfunction, we performed digital spatial profiling (DSP, GeoMx) of tumour-infiltrating myeloid cells (CD68) using a targeted immunobiology panel (78 genes). Myeloid cells were chosen as they were the second most dominant contributor to the RPLS signature, pDCs were enriched in RP TCs and myeloid cells can promote chemosensitivity independent of adaptive immune cells.^27^ In LS TCs, myeloid cells expressed high levels of cytotoxic effectors (*BCL2*, *GZMB*, *NKG7*, *TNF*) and cytokines (*CCL5*, *CXCR6*, *IL12B*, *IL15*, *TNF*) (figure 3F). RP tumour-infiltrating myeloid cells expressed high levels of molecules associated with immunosuppression (*CD58*, *CD80*, *CD163*), as well as monocyte activation and dendritic cell maturation (CD40). Consistent with failed immunogenic clearance, Ki-67 staining indicated tumour cells were proliferating faster in RP TCs (p=0.02; figure 3G). Altogether, these data implicate pretreatment antitumour immunity as a characteristic required for chemotherapy benefit.

Myeloid cells are highly diverse, so we analysed myeloid-specific RPLS scores (13 RPLS signature genes with predicted myeloid origin by digital cytometry) in scRNA-seq data generated from immune cells (CD45+) of 3 iCCA patients undergoing tumour resection.^28^ Under adjuvant capecitabine, one patient exhibited short disease-free survival (9 months; S-DFS) and two others exhibited long disease-free survival (L-DFS; ≥24 months) (figure 3H). Among the eight identified cell subpopulations, CD14 monocytes were more abundant in the L-DFS cases (p=0.03). However, evaluation of myeloid-specific RPLS scores identified increased signature expression across diverse myeloid cell types in S-DFS (CD140^+^ monocytes, ID3^+^ macrophages, MARCO^+^ macrophages, TREM2^+^ macrophages, CD1C^+^ cDC2, lymphoid-like cells). This implicates widespread behavioural changes of diverse myeloid subpopulations with diminished chemotherapy outcome.

### RP-like and LS-like iCCA are dependent on unique gene networks for survival in vitro

In patient biopsies, the dominant origin of the RPLS signature is the tumour cells. To determine whether immortalised iCCA cell lines recapitulate aspects of these tumour-intrinsic programmes, we integrated the transcriptome profiles of 25 iCCA cell lines with transcriptome data from our biopsy TCs (online supplemental figure S7A), annotating 52% (13/25) of cell lines as RP-like and the remainder as LS-like (online supplemental figure S7B). RP-like cells had decreased in vitro gemcitabine sensitivity (p=0.02; Figure S7C), trended towards association with *KRAS* mutations (p=0.07) (online supplemental figure S7D), and differentially expressed pathways (KRAS and P53 pathways, glycolysis) compared with LS-like cells (online supplemental figure S7E). These observations suggest that immortalised iCCA cell lines can provide minimalistic avatars to study some tumour-intrinsic aspects of RP-like and LS-like patient phenotypes in vitro. As chemoresistance is associated with distinct biology, RP-like phenotypes should also be associated with fitness tradeoffs, specifically genes which become more or less important for tumour cell survival. Using genome-wide CRISPR inactivation data (DepMap), we identified differential gene dependencies (Wilcoxon p<0.05) between RP-likeand LS-like iCCA that fell into common biological networks (online supplemental table S3). RP-like iCCA was more dependent on 48 network-based genes for survival (Notch, p53 and TGF-β signalling; online supplemental figure S7F), whereas LS-like iCCA was more dependent on 62 network-based genes (Hedgehog, Ras signalling; online supplemental figure S7G). Approximately 44% (21/48) and 26% (16/62) of these genes are predicted to be potentially druggable for RP-like and LS-like phenotypes, respectively (online supplemental table S3). These subgroup-specific dependencies indicate that considerable drug development opportunities remain for patients with iCCA, including those with RP-like phenotypes on standard-of-care chemotherapy.

10.1136/gutjnl-2023-330748.supp9 Supplementary data

### RP-like tumour cells engage immunosuppressive microenvironments via myeloid and T cell communication

**Figure 4:**
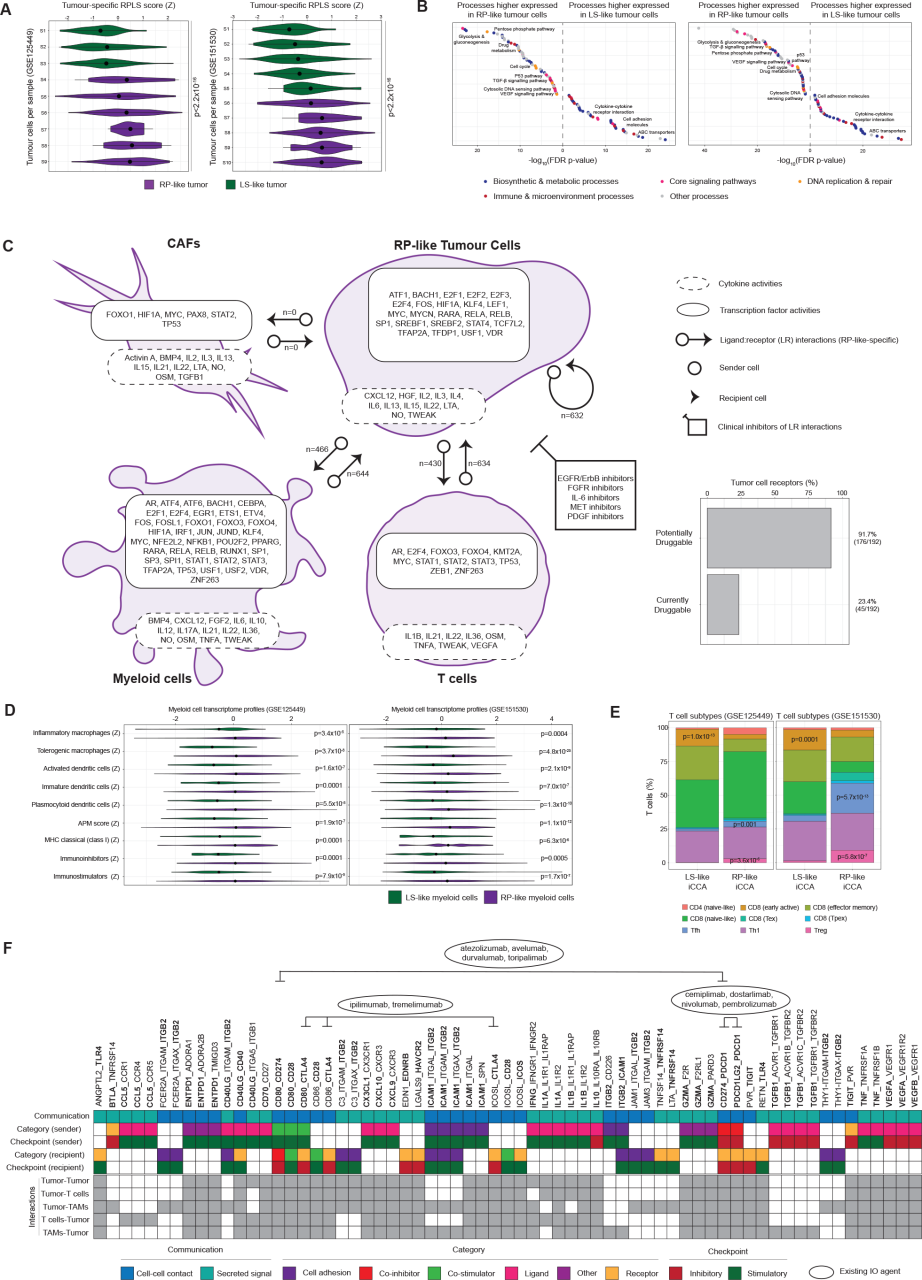
Modelling the RPLS signature in iCCA single cell RNA-sequencing data. (A) Annotation of tumours as long survivor (LS)-iike or rapid progressor (RP)-like based on tumour cell expression of the tumour-origin RPLS signature (ESCAPE tool, p values derived by Wilcoxon test). (B) Differentially expressed pathways and processes (ESCAPE with KEGG and Hallmarks gene lists) between LS-like and RP-like tumour cells. (C) Cell type-specific transcription factor activities (DoRothEA), cytokine activities (CytoSig) and ligand:receptor interactions (CellChat) unique to RP-like tumours in GSE125449 and GSE151530, including the potential and current druggability of tumour surface receptors. (D) Differential expression of myeloid cell type and functional signatures in LS-like and RP-like myeloid cells (ESCAPE, Wilcoxon test). (E) T cell subtype annotation using ProjectTILs (p values from Fisher’s exact test). (F) RP-specific ligand-receptor interactions tumours in GSE125449 and GSE151530 involving immunomodulatory targets (highlighted in bold; defined by CRI iAtlas). iCCA, intrahepatic cholangiocarcinoma.

Although in vitro models could partially recapitulate RPLS-associated oncogenic programmes, they lack interacting microenvironmental cells that are pronouncedly reprogrammed between RP and LS patient biopsies. Using two scRNA-seq iCCA datasets, we annotated patient tumours as RP-like or LS-like (figure 4A; Methods). Tumour cells mirrored pathway expression as observed in the immortalised cell lines, including elevated cytosolic DNA sensing, glycolysis, gluconeogenesis, and P53 pathways in RP-like tumours (figure 4B, online supplemental table S4). RP-like and LS-like tumour cells were also characterised by unique cytokine and TF activity profiles (figure 4C, online supplemental table S5), highlighting stable cell behavioural states. Microenvironment cells (CAFs, myeloid, T cells) also exhibited unique cytokine and TF activity profiles that consistently differed between RP-like and LS-like tumours (figure 4C, online supplemental table S5). Metabolic flux modelling identified increased methionine and losartan utilisation by RP-like myeloid cells, metabolites that are required for anti-tumour immunity^29 30^ but appear to be otherwise restricted by the myeloid compartment, potentially indicating metabolic competition as a contributory factor to immune dysfunction (online supplemental figure S8). To pinpoint tumour–microenvironment interactions that support these altered behavioural states, we identified ligand-receptor (LR) interactons (CellChat) unique to RP-like and LS-like iCCA. Whereas no LR interactions were unique to and reproducible for LS-like iCCA, RP-like iCCAs were characterised by many unique LR interactions: tumour-to-tumour (n=632), tumour-to-myeloid (n=466), myeloid-to-tumour (n=644), tumour-to-T cell (n=430), T cell-to-tumour (n=634) (figure 4C; online supplemental table S6). No unique interactions were found for CAFs, but this may be due to their under-representation in non-enriched scRNA-seq datasets. Receptors expressed on the surface of tumour cells have historically provided impactful therapeutic targets. Focusing on the RP-like tumour ‘surfaceome’ with evidence of LR interactions, roughly 92% (176/192) are predicted to be potentially druggable, whereas 23% (45/192) are currently actionable with clinically approved compounds (online supplemental table S7). These agents include IL-6R monoclonal antibodies which have been shown to improve chemotherapy response in a subcutaneous transplant model of iCCA,^31^ as well as several FGFR inhibitors already approved for second-line use in iCCA.^32^

10.1136/gutjnl-2023-330748.supp10 Supplementary data

Variation in microenvironment behavioural states might reflect differences in abundance of cell subpopulations. RP-like myeloid cells had higher signature expression for dendritic cells (figure 4D), including plasmocytoid dendritic cells that were increased in RP TCs (figure 3C), as well as inflammatory and immune tolerogenic subtypes of liver-associated macrophages. RP-like myeloid cells were defined by higher antigen expression scores (including classical MHC presentation), but also co-occuring expression of immunostimulator (including CD275 and IL6) and immunoinhibitor (including CSF1R, CTLA4, IDO1, LAG3, PDCD1) signatures (figure 4D). Subclassification of T cells into their functional ontogenies identified more regulatory T (Tregs) and T follicular helper cells in RP-like tumours, and a reduced amount of early active CD8 T cells (figure 4E). Collectively, these findings implicate tumour-induced immune tolerogenicity as a hallmark of RP phenotypes, defined by the activity of immunosuppressive cytokines (especially IL10^33^), antigen presentation in the presence of diverse immunoinhibitors and accumulation of regulatory T cells.

Combining chemotherapy with the PD-L1 inhibitor, durvalumab or the PD-1 inhibitor, pembrolizumab, improved OS of patients with BTC in the TOPAZ-1 trial^5^ and KEYNOTE-966 trial,^6^ respectively. Combination chemotherapy-immunotherapy represents the new standard-of-care, although mechanistic explanations and biomarkers for patient selection are lacking. By investigating LR interactions involving immunomodulatory targets (CRI-iAtlas), we identified interactions between CD274 (PD-L1), its receptor PDCD1 (PD-1) and its costimulator CD80 (B7-1) exclusively in RP-like tumours (figure 4F). These observations suggest that the immune escape mechanisms employed by RP-like tumours might render them susceptible to checkpoint inhibitors compared with LS-like tumours. Other RP-specific targets for potential immunomodulatory inhibitors include immunosuppressive cytokines (IL10), ligands (PDCD1LG2, TGFB1, VEGFA, VEGFB) and receptors (EDNRB).

### The RPLS signature is prognostic and pathobiologically distinct in early-stage iCCA

**Figure 5:**
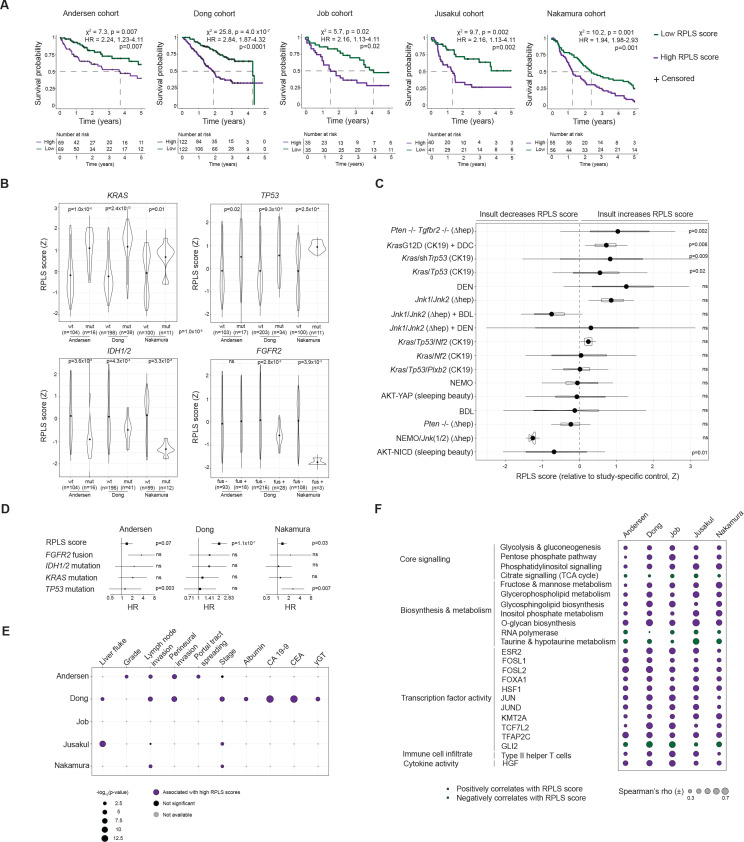
Prognostic, clinicogenomic and transcriptomic associations of the RPLS signature in 653 resected iCCA. (A) Kaplan-Meier survival curves with log-rank statistics of resected iCCA stratified into high (>median) or low (<median) RPLS score groups across five resected cohorts. (B) Differential expression (Wilcoxon test) of RPLS scores between iCCA stratified by genotype across three cohorts where RNA-profiling and DNA-profiling data are available. (C) Differential expression (Welch t-test) of RPLS scores between mouse models of iCCA and their study-specific controls. Cell type-specific induction of genetic insults are indicated as CK19 (biliary/progenitor cell) or hep (hepatocyte). ∆: deletion; (D) Forest plot of Cox proportional hazards statistics derived from multivariable analysis of RPLS scores and tumour genotypes. (E) Associations of RPLS scores with clinicopathological variables. γ-GT: γ-glutamyltransferase; CA 19–9: carbohydrate antigen 19–9; CEA: carcinoembryonic antigen. (E) Forest plot depicting ORs and p values from multivariable analysis of RPLS scores, tumour stages and genotypes across three resected cohorts. (F) Correlation plot of RPLS score with core signalling pathways (KEGG), biosynthesis and metabolic processes (KEGG), transcription factor activity (DoRothEA), immune cell infiltrate (xCell) and cytokine activity (CytoSig) across five resected cohorts. Spearman’s r is only indicated for significantly correlated features (FDR p<0.05). BDL, bile duct ligation; DDC, 3,5-diethoxycarbonyl-1,4-dihydrocollidine; DEN, diethylnitrosamine; FDR, false discovery rate; iCCA, intrahepatic cholangiocarcinoma; mut, mutant; ns, not significant; wt, wild-type.

Although the RPLS signature was identified in advanced iCCA biopsies, its origin in tumour-immune interactions suggests that the determinants of chemotherapy response might be established early during cholangiocarcinogenesis. Therefore, we investigated the RPLS signature in 637 fresh-frozen iCCA.^19 34–37^ As with our biopsy cohort of advanced patients, LS-high and RP-high genes were anticorrelated in all resected cohorts, emphasising the reproducibility of the observed inter-network signalling (online supplemental figure S9). High RPLS scores (above median) were consistently associated with decreased 5-year survival in all cohorts (figure 5A). High RPLS scores were also associated with inferior survival in an FFPE cohort of 119 iCCA,^38^ but not in 219 extrahepatic CCA^39^ (online supplemental figure S10). As such, the RPLS signature appears to be prognostic exclusively in iCCA, further suggesting the importance of the liver microenvironment in the RPLS signature. Data on adjuvant or subsequent palliative therapy are not available and therefore association with chemoresistance cannot be deducted.

10.1136/gutjnl-2023-330748.supp11 Supplementary data

10.1136/gutjnl-2023-330748.supp12 Supplementary data

RPLS scores were lower in tumours harbouring *IDH1 or IDH2* mutations (all 3 cohorts with data available), as well as *FGFR2* fusions (2/3 cohorts) (figure 5B). Conversely, tumours with *KRAS* or *TP53* mutations had higher RPLS scores in all cohorts, indicative of a higher baseline level of innate chemoresistance. Evaluation of 17 mouse models of iCCA revealed RPLS scores to become elevated in 24% (4/17) of model tumours relative to their controls, among which three involved insults in *Kras* and/or *Tp53* (figure 5C). RPLS scores were also increased in intraductal papillary neoplasm of the bile duct relative to ductular proliferation and normal tissues in cholangiocyte-specific *Kras* ^G12D^-expressing mice (online supplemental figure S11A), suggesting that RPLS-associated chemoresistance is established early during cholangiocarcinogenesis. Combining RPLS scores with associated genomic alterations in multivariable models revealed the RPLS signature to consistently provide genotype-indepedent prognostic information across cohorts (figure 5D).

10.1136/gutjnl-2023-330748.supp13 Supplementary data

Clinically, RPLS scores were consistently higher in iCCA with liver fluke infection, advanced grade, perineural involvement and portal tract spreading (figure 5E; online supplemental table S8). RPLS scores were higher in tumours with advanced stage and lymph node invasion (3/4 cohorts with data available) and positively correlated with serum albumin, CA19-9, CEA and GGT (figure 5E). Higher RPLS scores were associated with large duct-type iCCA in a small reseceted cohort (p=0.007; GSE107943; online supplemental figure S11B), consistent with our mutation observations (*KRAS* and *TP53*) (figure 5B) and previous associations between morphology and chemotherapy outcome.^40^ In the Dong cohort where transcriptome and clinicopathological data are present for all patients, the RPLS signature is an independent prognostic variable after correcting for its clinicopathological correlates (online supplemental figure S11C), highlighting the potential utility of this metric in the resected setting where surgical and post-surgical specimen evaluation is possible (unlike the advanced setting).

Transcriptomically, the RPLS signature was associated with expression of metabolic pathways (glycolysis and gluconeogenesis, pentose phosphate pathway, phosphatidylinositol signalling, citrate signalling) (figure 5F). The RPLS signature was also reproducibly associated with key TF activities, immune cell infiltrates and cytokine activities. Therefore, RPLS-associated oncogenic programmes exhibit robust pathobiological associations and reflect a significant source of intertumour heterogeneity.

### The RPLS signature captures a liver-specific oncogenic programme and predicts chemotherapy outcome in liver-metastatic colorectal cancer

**Figure 6:**
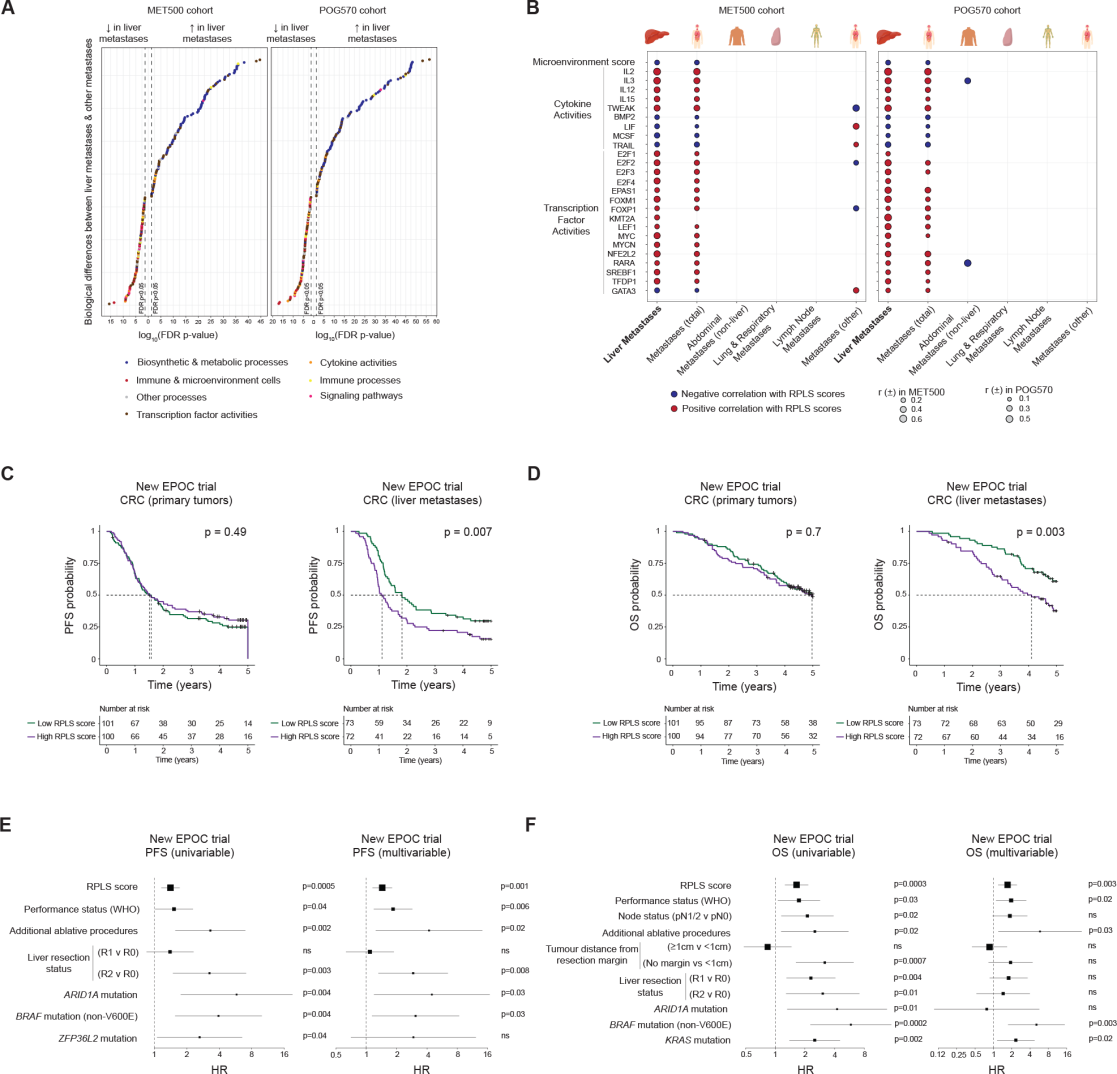
Pathobiological associations and predictive potential of the RPLS signature in liver metastases. (A) Differential expression of pathways and processes (ssGSEA with KEGG and Hallmarks gene lists) between liver metastases and other metastases in MET500 (n=490) and POG570 (n=438) cohorts (p values derived from Wilcoxon test). (B) Biological processes uniquely associated with the RPLS signature in liver metastases. (C, D) Kaplan-Meier survival curves with log-rank statistics of primary colorectal cancer tumours (n=204) and liver metastases (n=145) stratified by RPLS score (above and below median) for (C) progression-free survival and (D) overall survival in the New EPOC trial. (E–F) Univariable and multivariable Cox proportional hazards analysis of RPLS scores and other significant clinicogenomic variables for (E) progression-free and (F) overall survival. ns, not significant.

Our observations that the RPLS signature is prognostic in iCCA but not eCCA indicate that the liver microenvironment plays an important role. As we found the RPLS signature is not prognostic in HCC (online supplemental figure S12) which is treated using targeted therapy-based regimens, we hypothesised that the RPLS signature is predictive for primary or metastatic liver tumours treated with chemotherapy. Accordingly, we investigated the RPLS signature in two basket cohorts of metastatic cancers: MET500 (n=484) and POG-570 (n=438).^41 42^ Consistent with the biological functions of the liver, liver metastases had higher expression of metabolic processes compared with other metastases in both cohorts (figure 6A, online supplemental table S9). These included glycolysis and gluconeogenesis, processes associated with increasing RPLS scores across iCCA models (advanced and resected patient tissues, immortalised cell lines, scRNA-seq). However, liver metastases were also remarkably depleted in diverse immune cells and immune processes compared with other metastatic sites, and associated with unique cytokine and TF activities, including a negative association with TRAIL activity as observed in iCCA biopsies (figure 6B). Exclusively in the liver, RPLS scores negatively correlated with microenvironment signalling, highlighting the extensive immunosuppressive capacity of the liver in coordination with specific oncogenic programmes.

10.1136/gutjnl-2023-330748.supp14 Supplementary data

Finally, we demonstrated the predictive utility of the RPLS signature in cancers with liver metastases undergoing chemotherapy. To evaluate this, we applied the RPLS signature to primary tumours and resected liver metastases from colorectal cancer patients receiving preoperative and postoperative chemotherapy with or without cetuximab in the phase III New EPOC trial.^43^ In the total cohort composed of both treatment arms, RPLS scores were not associated with progression-free survival (PFS; p=0.49) or OS (OS; p=0.7) when evaluated in primary tumours (figure 6C,D). However, high RPLS scores were associated with inferior PFS (p=0.007) and OS (p=0.003) when measured in liver metastases. Liver metastasis RPLS scores were predictive of PFS (HR 1.4, 95% CI 1.2 to 1.7; online supplemental table S10) and OS (HR 1.6, 95% CI 1.3 to 2.2; online supplemental table S11) in univariable analyses in the New EPOC trial (figure 6E). These RPLS scores remained independent predictors of PFS (HR 1.4, 95% CI 1.2 to 1.8) and OS (HR 1.7, 95% CI 1.2 to 2.4) after adjusting for clinicopathologic and genomic predictors in multivariable analyses (figure 6F). Based on the robust predictive performance of the RPLS signature in primary (iCCA) and metastatic (colorectal) liver tumours treated with chemotherapy, continued clinical evaluation of this metric is warranted.

## Discussion

**Figure 7:**
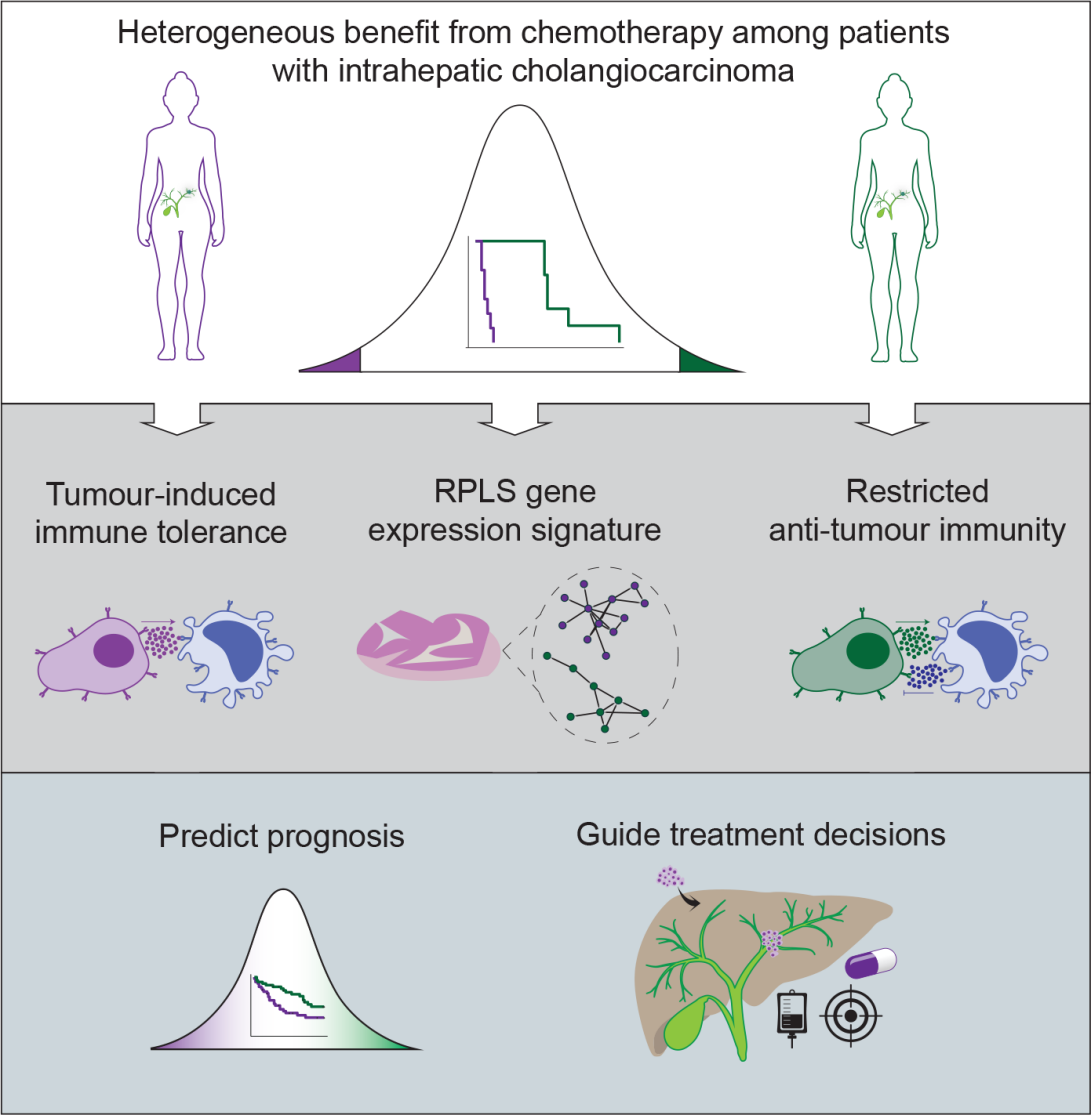
Graphical schematic of RPLS-associated chemosensitivity in iCCA. Heterogeneous benefit from chemotherapy is associated with tumour-induced tolerogenicity and restricted anti-tumour immunity. Pending further validation, the RPLS signature could clinically empower accurate prognostic prediction and guide treatment selection. iCCA, intrahepatic cholangiocarcinoma.

Heterogeneous benefit from chemotherapy challenges the paradigm of this universal standard-of-care for patients with iCCA. In this study, we aimed to identify the biology associated with different clinical outcomes in iCCA undergoing chemotherapy (figure 7). Based on our data, we hypothesise that multilayered mechanisms contribute to RPLS-associated chemoresistance, involving tumour-intrinsic processes, tumour-myeloid interactions and tumour-T cell interactions. Decreased in vitro sensitivity of RP-like cell lines likely originates from metabolic reprogramming such as increased glycolysis which has been shown to confer chemoresistance to cytotoxic agents in diverse cancers.^44 45^ In immunosuppressive microenvironments, macrophages can further limit the antitumour effects of chemotherapy, by metabolically inactivating gemcitabine prior to drug uptake by cancer cells^27^ and actively decreasing the duration of mitotic arrest of tumour cells following induction of DNA damage.^46^ Copresentation of tumour antigens alongside immunosuppressive molecules by myeloid cells promotes regulatory T cell expansion and tumour tolerogenicity, culminating in a steady state of immunological inertia. In this context, the unique immunoregulatory capabilities of the liver microenvironment appear to be critical, replete with atypical dendritic cells that normally function to suppress systemic immune responses arising from continuous exposure to antigens and gut microbial byproducts. Such systemic regulation of immunity by the liver may explain why only RPLS scores from liver tumours (primary and metastatic) are associated with chemotherapy outcome in our studies. A similar phenomenon has been reported for immunotherapy, in which liver metastases uniquely blunt checkpoint inhibitor response through hepatic macrophage-mediated elimination of CD8 T cells.^47^ A critical question emerging is why only certain tumours in the liver can trigger high RPLS scores leading to diminished chemotherapy response. One commonality across in vitro and ex vivo analyses was high glycolytic pathway expression in models with high RPLS scores. Increased glucose consumption and lactic acid production are associated with immunosuppressive microenvironments,^48^ and myeloid cells are the highest cellular consumers of glucose,^49^ potentially establishing a metabolically initiated and competitive tumour niche.

Advancing the RPLS signature into a clinical grade test will require further optimisation in large retrospective cohorts. This will include statistically optimising the signature into a smaller gene panel with a weighted formula and establishing reference value ranges for interpretation of individual patient risk (radiological response, PFS, OS) if treated with chemotherapy. Pending continued validation, clinical implementation of the optimised RPLS signature could prioritise patients for neoadjuvant chemotherapy (high-risk resectable or borderline resectable) and support earlier tumour molecular profiling in predicted RP patients to identify alternative first-line treatment strategies. For predicted RP patients currently lacking alternative treatment strategies, further therapeutic evaluation of RP-associated biology (Notch, TGF-β, IL-6, immune checkpoints) is warranted. A complementary transcriptome-driven approach will also be important to predict benefit from chemotherapy-immunotherapy combinations. Pursuing such a precision chemotherapy approach will be critical for optimising patient management and decision-making as the treatment landscape continues to evolve in the present and future.

10.1136/gutjnl-2023-330748.supp8 Supplementary data

## Data Availability

Data are available on reasonable request. Transcriptome data have been deposited in Gene Expression Omnibus (GEO) under this manuscript.
