# Supplementary material for: Molecular portraits of patients with intrahepatic cholangiocarcinoma who diverge as rapid progressors or long survivors on chemotherapy
Source: Gut. 2023 Sep 27;73(3):496–508. doi: 10.1136/gutjnl-2023-330748 (PMC10894814; doi:10.1136/gutjnl-2023-330748)
Supplement: Supplementary data [file gutjnl-2023-330748supp001.pdf]

Molecular portraits of patients with intrahepatic cholangiocarcinoma who diverge as rapid progressors or long survivors on chemotherapy

Colm J O'Rourke[1*], Massimiliano Salati[2,3*], Colin Rae C[4], Guido Carpino[5], Holly Leslie[4], Antonio Pea[4], Maria G Prete[4], LR Bonetti[6], F Amato[4], R Montal[7], R Upstill-Goddard[4], Colin Nixon[8], Paula Sanchon-Sanchez[4], Paolo Kunderfranco[9], Daniela Sia[10], Eugenio Gaudio[5], Diletta Overi[5], Stefano Cascinu[11], Dan Hogdall[1,12], Sian Pugh[13], Enric Domingo[14], John N Primrose[15], John Bridgewater[16], A Spallanzani[2], F Gelsomino[2], JM Llovet[17,18,19], Diego F. Calvisi[20], Luke Boulter[21,22], F Caputo[2], Ana Lleo[23,24], Nigel B Jamieson[4,22], G Luppi[2], M Dominici[2], Jesper B Andersen[1*#], Chiara Braconi[4, 22, 25*#]

[1]Biotech Research and Innovation Centre (BRIC), Department of Health and Medical Sciences, University of Copenhagen, Copenhagen N, Denmark.
[2]Division of Oncology, Department of Oncology and Hematology, University Hospital of Modena, Modena, Italy.
[3]PhD Program, Clinical and Experimental Medicine, University of Modena and Reggio Emilia, Modena, Italy.
[4]School of Cancer Sciences, University of Glasgow, Scotland, United Kingdom.
[5]Department of Anatomical, Histological, Forensic Medicine and Orthopaedic Sciences, Sapienza University of Rome, Rome, Italy.
[6]Division of Pathology, University of Modena and Reggio Emilia, Emilia-Romagna, Rome, Italy.
[7]Cancer Biomarkers Research Group, Department of Medical Oncology, Hospital Universitari Arnau de Vilanova-IRBLleida, Lleida, Catalonia, Spain.
[8]CRUK Beatson Cancer Research Institute, Glasgow, UK.
[9]Bioinformatics Unit, IRCCS Humanitas Research Hospital, Rozzano, Milan, Italy.
[10]Liver Cancer Program, Divisions of Liver Diseases, Pathology Department and RM Transplant Institute, Tisch Cancer Institute, Department of Medicine, Icahn School of Medicine at Mount Sinai, New York, New York, USA.
[11]Department of Medical Oncology, IRCCS San Raffaele Hospital, Milan, Italy.
[12]Department of Oncology, Herlev and Gentofte Hospital, Herlev, Copenhagen University Hospital, Copenhagen, Denmark.
[13]Addenbrooke's Hospital, Cambridge, United Kingdom.
[14]Department of Oncology, University of Oxford, Oxford, England, United Kingdom.
[15]Department of Surgery, University of Southampton, Southampton, United Kingdom.
[16]UCL Cancer Institute, London, United Kingdom.
[17]Translational Research in Hepatic Oncology, Liver Unit, IDIBAPS, Hospital Clínic, University of Barcelona, Barcelona, Catalonia, Spain.
[18]Liver Cancer Program, Divisions of Liver Diseases, Pathology Department and RM Transplant Institute, Tisch Cancer Institute, Department of Medicine, Icahn School of Medicine at Mount Sinai, New York, New York, USA.
[19]Institució Catalana de Recerca i Estudis Avançats (ICREA), Barcelona, Catalonia, Spain.
[20]Institute of Pathology, University of Regensburg, 93053 Regensburg, Germany.

[21]MRC Human Genetics Unit, Institute of Genetics and Cancer, University of Edinburgh, Edinburgh, UK.
[22]Cancer Research UK Scotland Centre, Glasgow-Edinburgh UK.
[23]Department of Biomedical Sciences, Humanitas University, Pieve Emanuele, Milan, Italy.
[24]Internal Medicine and Hepatology Unit, Department of Gastroenterology, IRCCS Humanitas Research Hospital, Rozzano, Milan, Italy.
[25]Beatson West of Scotland Cancer Centre, Glasgow, UK.
*Equal contribution, [#]corresponding authors

SUPPLEMENTARY INFORMATION

Total number of items: 12 supplementary figures, 11 supplementary tables

SUPPLEMENTARY MATERIALS & METHODS

RPLS COHORT

Patient selection

Patients with histologically proven advanced iCCA treated with first-line chemotherapy were retrospectively identified from the Modena Cancer Centre Biliary Tract Cancer Database, after review from the appropriate health research authorities (BILONG study protocol 465/18 - reviewed by the Area Vasta Emilia Nord Ethics committee). Patients were deemed eligible if they presented with *de novo* advanced unresectable iCCA (i.e. locally advanced or metastatic) and tissue from diagnostic liver biopsies were available. Patients with a diagnosis of mixed iCCA/HCC were excluded. Neither prior surgery nor liver-directed treatment were allowed. Clinical and laboratory data were retrieved retrospectively through electronic medical records review. The following baseline variables were collected and analysed before the commencement of first-line chemotherapy: age, sex, Eastern Cooperative Oncology Group (ECOG) performance status (PS), primary tumour site, disease status, location of metastatic sites. Overall survival (OS) was defined as the time from the first dose of first line chemotherapy to death. To meet the definition of rapid progressor (RP), patients had to have an OS ≤ 6 months which is below half the median survival time reported by the ABC-02 trial [1]. Long survivors (LS) were defined those patients who had OS ≥23 months since starting of chemotherapy, more than double the median survival time reported by the ABC-02 trial. The two patient subgroups were matched for major clinical features. None of these patients had

background liver cirrhosis. The study protocol conformed to the ethical guidelines of the 1975 Declaration of Helsinki.

Human tissues

All patients underwent ultrasound-guided liver biopsy before the commencement of systemic treatment. Formalin Fixed Paraffin Embedded (FFPE) tissue slides were retrieved from liver biopsy. One slide was processed for targeted sequencing-based RNA expression analysis via TempO-Seq (Bioclavis, Glasgow, UK). One slide was used for macroscopic dissection of specific ROIs identified through a pathology review; tissue from each (region-of-interest) ROI was then subjected to targeted sequencing-based RNA expression analysis via TempO-Seq (Bioclavis, Glasgow, UK). A third consecutive slide was used for Digital Spatial Transcriptomic (see below).

Digital pathology

Hematoxylin-eosin slides were digitized using the APERIO platform (Leica Biosystems) at 20X of magnification. Each slide was analysed using the open-source software platform, QuPath (version 0.2.3) [2]. A region of interest (ROI) was annotated for each slide and the amount of tumoural tissue quantified. First, we characterised the amount of epithelial and stromal tissue components in each ROI by generating a random tree forest pixel classifier. Second, following cell detection on each ROI, a different random tree forest cell classifier was generated for each haematoxylin-eosin slide using cell features to classify cells into tumour, immune and stromal cells. To help the algorithm to perform an accurate classification, smoothed features at 25 radii were added and multiple rounds of cell classification review were performed.

Whole-transcriptome profiling by Tempo-seq

Whole transcriptome gene expression analysis was performed using the TempO-Seq Human Whole Transcriptome v2.0 panel (BioClavis, Glasgow, UK). Internal quality control was performed according to the following criteria: 1) number of mapped reads in positive RNA controls > 6 million [our study: 6,703,241]; 2) signal:noise ratio as the ratio between the total number of reads in the positive control and the total number of reads in the negative controls

>20 [our project 205;1]; 3) the percentage of mapped reads in positive controls > 80% [our project 97%]; 4) average reads/probe >250 [our project 471]. Process controls were run in replicate on each assay plate of samples to ensure quality metrics pass on a plate-wise level. High reproducibility of positive controls, and low signal in the negative control was observed in our project.

Sequencing libraries for targeted panels were generated; briefly in TempO-Seq, each Detector Oligo consisted of a sequence complementary to an mRNA target plus a universal primer binding site. They annealed in immediate juxtaposition to each other on the targeted RNA template such that they can be ligated together. Ligated detector oligos were PCR-amplified using a primer set (single-plex PCR reaction, with a single primer pair for each sample) that introduced both the adaptors required for sequencing and a sample-specific barcode. The barcode sequences flanked the target sequence and were inserted appropriately into the standard Illumina adaptors to permit standard dual-index sequencing of the barcodes and deconvolution of sample-specific reads from the sequencing data using the standard Illumina software. All the PCR-amplified and barcoded samples were pooled into a single library for sequencing. Sequencing reads were demultiplexed using the standard sequencing instrument software for each sample using the barcodes to give a FASTQ file for each.

TempO-Seq sequence files were analysed using the Tempo-SeqR software package. Each FASTQ file was aligned using the STAR algorithm to a pseudotranscriptome corresponding to the gene panel used in the assay. Data were normalized using DESeq2 [3]. Transcriptome data can be made available upon request following IRB approval.

GeoMx digital spatial profiling

NanoString GeoMx digital spatial profiling

To further characterise variation in transcriptomic expression between tumour-infiltrating myeloid cells in LS and RP patients, formalin-fixed paraffin-embedded (FFPE) sections of 12 cholangiocarcinoma biopsies were selected for analysis on the NanoString GeoMx Digital Spatial Profiler (DSP). This platform enables the characterisation of user-selected topographic Regions of Interest (ROI) from immunofluorescently (IF) stained FFPE tissue. The GeoMx instrument achieves RNA profiling in situ hybridization by employing DNA oligonucleotide

probes designed to bind mRNA targets. From 5′ to 3′, they comprise a 35- to 50-nucleotide target complementary sequence, an ultraviolet (UV) photocleavable linker and a 66-nucleotide indexing oligonucleotide sequence containing a unique molecular identifier (UMI), RNA ID sequence and primer binding sites. Up to 10 RNA detection probes were designed per target mRNA. In summary, the instrument employs UV light to cleave the UV-sensitive probes leading to release of the hybridized barcodes.

Slide preparation including hybridization of tissue with UV-photocleavable probes

The DSP procedure has previously been described in detail by Merritt et al [4] with our own protocol described in Fisher et al [5]. 5-µm FFPE sections of the 12 cholangiocarcinoma biopsies were mounted on positively charged Superfrost glass slides (ThermoFisher Scientific) and baked for 1hr at 60°C. The tissue was dewaxed, hydrated and treated with 1 µg/ml Proteinase K (ThermoFisher Scientific, AM2546) for 15 minutes before undergoing heat-induced epitope retrieval (HIER) on a Leica BOND Autostainer (pH 9.0, ER2 at 100°C) for 20 minutes. The slides were then stored until required in 1X PBS (PBS: Invitrogen, AM9625).

In-situ hybridization of RNA-directed DNA oligo probes (Immune Pathways Panel included 96 genes with the addition of CD58, ELK4, CD80, CD163, FOXO3, NanoString) was performed as per manufacturer’s protocol. HybriSlip™ covers were applied prior to overnight incubation at 37°C for at least 16 hours (ThermoFisher). The following day, slides were washed twice with a 1:1 ratio of 100% deionized formamide (Ambion) and 4X SSC (Sigma Aldrich) at 37°C for 25 minutes.

The GeoMx DSP is capable of capturing four channels (FITC/525nm, Cy3/568nm, Texas Red/615nm and Cy5/666nm) for the detection of up to four customizable IF morphology markers for each tissue [4]. One channel (FITC/525nm) is reserved for the nuclear stain (SYTO13). The slides were blocked with Buffer W (Nanostring) for 30 minutes at RT before incubation. Immunofluorescence staining was performed using primary conjugated antibodies (PanCK (NanoString), CD68 (clone KP1, 1:200, Santa Cruz) and Ki67 (clone D2H10, 1:100, Cell Signaling Technologies)) and nucleic acid dye (SYTO 13 (NanoString)) as per the manufacturer protocol. Slides were then stored at 4°C in SSC before being loaded on the GeoMx DSP instrument for region of interest (ROI) selection and collection.

Region selection and collection

The whole slides were imaged at 20x magnification using the GeoMx DSP, with the integrated software suite used to select ROIs for downstream analysis based on the 4-plex immunofluorescence staining of SYTO 13, PanCK, CD68 and Ki67. Polygonal ROIs were selected primarily according to the high density of CD68+ cells. 30 ROIs were selected in total from across the 12 slides. The CD68 staining was then used to employ segmentation within each ROI to create an area of interest (AOI) from which DNA oligo probes would be cleaved and cell-type specific transcriptomic profiles obtained. After AOIs were selected, the GeoMx DSP employs an automatically controlled UV laser (385nm) to illuminate each AOI in turn, specifically cleaving barcodes within the CD68+ immunofluorescent (IF) segments but not in surrounding tissue segments (CD68-). A microcapillary collection system collected the liberated barcodes from each AOI and plated them into individual wells on a 96-well microtiter plate. This process was repeated in turn for each AOI before downstream processing using the NanoString MAX/FLEX nCounter system. The oligonucleotides were dried overnight and subsequently resuspended using 7 µl of DEPC-treated water.

nCounter hybridization assay for photocleaved oligo counting

The nCounter readout of GeoMx DSP-collected probes was performed according to manufacturer's protocol (NanoString, MAN-10089-08). In brief, samples were resuspended in dH20 prior overnight incubation (16–24 hours) with hybridization codes (Hyb Codes) at 65°C and heated lid (70°C). These Hyb Codes include reporter and capture probes to enable formation of a tripartite hybridization complex with the DNA oligo probes in the panel. Samples were then pooled by column into a 12-well strip tube before processing on NanoString's MAX/FLEX system, using the high sensitivity protocol (NanoString, MAN-10089-08).

Data processing and analysis

Data acquisition was performed by using the NanoString's Digital Analyzer (field of view, 555) and Digital Count Conversion (DDC) files were re-uploaded onto the GeoMx DSP for mapping of transcriptomic data to the spatial origin, where they underwent quality control, filtering normalization and background correction.

Bioinformatic analyses

RPLS signature identification

RPLS signature genes were identified as genes differentially expressed between RP and LS patients with ≥ 2-fold expression difference and unadjusted $p<0.05$ (Wilcoxon rank-sum test). As RP-high and LS-high signature genes significantly anti-correlated, a single RPLS score was assigned to each tissue sample, defined as: ($[\log_2(\Sigma\text{RP-high genes}) - \log_2(\Sigma\text{LS-high genes})]_{z\text{-score}}$). To compare the predictive performance of the RPLS score against systemic features, it was included into multivariable Cox proportional hazards models with pre-treatment systemic features that differed between patient subgroups (platelets, ALP) or an optimized systemic signature (defined by AIC backwards elimination using all haematological and systemic features). The final formula derived for this optimized signature was: -211.622 + 41.2*Bili + 1.208*ALP.

Pathway analysis

Pathway over-representation analysis of RPLS signature genes was performed using ENRICHR against the Kyoto Encyclopedia of Genes and Genomes (KEGG) database [6] (including censoring of the non-relevant categories, “infectious disease” and “substance dependence”). Significantly over-represented pathways ($p<0.05$) were visualized using CytoScape (v3.9) [7]. Specifically, the EnrichmentMap application[8] was used to construct network maps in which each node represents a significant pathway and each line denotes shared genes between pathways (Jaccard coefficient above 0.375). Inter-related pathways were identified and biologically classified by Markov Cluster Algorithm (MCL) using the AutoAnnotate application (v1.3.3) [9].

Pathway expression analysis was performed using single sample gene set enrichment analysis (ssGSEA), as implemented in the ‘GSVA’ R package [10]. Prior to ssGSEA, transcriptome data were centered and scaled. KEGG [6] and Hallmarks [11] terms were downloaded from Molecular Signatures Database (MSigDB) v7.5.1 [12, 13] and used as input gene lists for ssGSEA. Only human- and cancer-relevant gene list categories were considered.

Transcription factor activities were predicted from transcriptome data using DoRothEA [14]. A gene was predicted to be under regulation of a specific transcription factor if it is a known transcription factor [14] and if its expression positively correlates with activity of the candidate transcription factor regulator.

Gemcitabine resistance genes and signatures

The expression of four genes involved in gemcitabine transport and metabolism that have been previously implicated in gemcitabine sensitivity [15, 16, 17] were investigated in our study: human equilibrative nucleoside transporter-1 (hENT1, encoded by *SLC29A1*); deoxycytidine kinase (*dCK*); ribonucleotide reductase catalytic subunit M1 (*RRM1*); ribonucleotide reductase catalytic subunit M1 (*RRM1*).

Three gene expression signatures previously reported to predict gemcitabine sensitivity were also evaluated in our study:

Mourragui et al. signature: This signature was identified based on *in vitro* sensitivity of 1,000 immortalized cancer cell lines to gemcitabine [18]. It consists of a weighted linear sum of expression of 1774 genes which was applied to each bulk tissue sample in the RPLS cohort.

Nicolle et al. signature: Also known as GemPred, this signature was identified based on *in vitro* sensitivity of 38 primary pancreatic adenocarcinoma (PDAC) cell lines to gemcitabine [19]. Whole-transcriptome profiles of RPLS samples were submitted to the GemPred web application (http://cit-apps.ligue-cancer.net/pancreatic_cancer/GemPred) in order to return a score per sample. Unreferenced values were used for further analysis.

Tiriac et al. signature: This signature was identified based on in vitro sensitivity of 77 PDAC organoids to gemcitabine [20]. It consists of 225 genes whose expression correlates (95 positively, 130 negatively) with gemcitabine sensitivity. These genes were combined into a weighted linear formula where each gene was assigned a coefficient proportional to its correlation coefficient, enabling assignment of an overall signature score to each of the bulk tissue samples in the RPLS cohort.

Digital cytometry

Cell type-specific deconvolution of the RPLS signature was performed in our RPLS cohort using CIBERSORTx [21]. Briefly, two public single cell RNA-sequencing (scRNA-seq) datasets from iCCA patients were downloaded from GEO (GSE125449 [22], GSE151530 [23]). These data were processed independently using the 'Seurat' package (v3) [24], including exclusion of outlier cells (mitochondrial content >0.05, gene counts <200 or 2500) and log normalization (scale factor of 10000). Six types of cells were commonly annotated by the original two scRNA-seq studies: B cells, cancer-associated fibroblasts (CAFs), T cells, tumour cells, tumour-associated myeloid cells (reannotated from cells originally reported as tumour-associated

macrophages), and tumour-associated endothelial cells (TECs). Only genes commonly detected in both datasets were used for gene expression classifier construction. Independently, a signature matrix was constructed for each scRNA-seq dataset in CIBERSORTx ('scRNA-Seq' as input data type with default settings). Each signature matrix was then applied independently to impute cell expression of the RPLS signature genes in our biopsy tissues (whole biopsies, tumour regions, tumour-associated stroma regions). This involved running the 'Impute Cell Expression' module of CIBERSORTx in 'Group Mode' with 'S-mode' batch correction and quantile normalization set as disabled. Each gene in the RPLS signature was only considered to originate from a specific cell type if this association was reproducible using both independent signature matrices (cell type-specific gene expression >1).

Immunogenomic and microenvironment analyses

Immune cell composition was predicted by cellular deconvolution using the xCell tool [25], implemented in the 'immunedeconv' package [26] with default settings. Anti-tumour immune activity was inferred using the cytolytic score, defined as the geometric mean of expression of granzyme A (*GZMA*) and perforin (*PRF1*) [27]. Cytokine activity was estimated using the CytoSig tool with default settings [28], implemented in Python on transcriptome data that was centralised and log2-transformed (including a pseudo-count of 1). Cancer testis antigen (CTA) expression was investigated using ssGSEA of CTA lists reported by CTdatabase (http://www.cta.lncc.br/) [29]. A gene expression-based microsatellite instability signature was calculated for RPLS samples using the Tumour Immune Dysfunction and Exclusion (TIDE) webtool [30]. A gene expression-based signature predictive of BRCA gene functionality and comprised of a weighted linear formula of 60 genes was used to assign a "BRCAness" score to each RPLS sample [31]. DNA repair signatures were obtained from Reactome [32]. Signatures associated with cancer-associated fibroblast subtypes detected in iCCA were investigated using ssGSEA with the gene lists reported in the original study [33].

IMMORTALIZED iCCA CELL LINES

Integration of cell line and RPLS tissue transcriptome data

Transcriptome data (RNA-seq) for 25 iCCA cell lines was downloaded from DepMap (22Q2) [34] quantified in log_2(TPM+1) units. In total, 14,728 genes were commonly detected in the cell line dataset and our RPLS tumour core (TC) dataset. Datasets were combined together by

performing quantile normalization ('PreProcess' package) followed by Combat batch correction ('sva' package) [35]. Removal of batch effects was confirmed by principal component analysis.

Classification of cell lines as LS- or RP-like

To develop a scoring method for RP- and LS-likeness, generalized linear models were run to identify genes individually capable of differentiating RP TCs from LS TCs in the adjusted dataset. In total, 441 significant genes (p<0.05) were identified. To generate an optimized classifier from these genes, least absolute shrinkage and selection operator (LASSO) regression was performed, resulting in the following classifier: -0.473260081235793 + (-0.20222060165566*CERS2) + (-0.0619806057536049*CHST9) + (-0.158248138046384*DCDC2) + (0.343556411268419*IGFBP4) + (-0.157768591678966*KPNA6) + (0.153671854832587*PDE12) + (0.041350560922029*PTPRU) + (-0.00927545715353832*SLC5A9) + (0.029840846853945*SPTLC3) + (0.105321982050334*STEAP2). This classifier was then applied to the adjusted cell line data to identify LS-like (< median; n=12) and RP-like (> median; n=13) cell lines.

Pharmacologic, genomic and transcriptomic comparison of LS- and RP-like cell lines

Gemcitabine sensitivity was compared between LS- (n=4) and RP-like (n=4) cell lines using previously reported high-throughput drug screening data from Saha and colleagues [36]. Differential expression of pathways and processes was compared between LS- and RP-like cell lines using ssGSEA, as earlier described (section 1.6.2). Hotspot mutation data were available for all 25 iCCA cells through DepMap (22Q2) [34]. Association of recurrent hotspot mutations (*IDH1*, *KCNA1*, *KIF4B*, *KRAS*, *PTEN*, *TBX5*, *TP53*) with LS- or RP-like cell lines was investigated by fisher's exact test for count data.

Identification of differential genetic dependencies

Genome-wide CRISPR inactivation screen data were available for 24 iCCA cell lines through DepMap (22Q2) [37], with gene effect scores inferenced by Chronos [38]. Pan-essential genes (n=2040) as reported by Dempster and colleagues [39] were filtered out from the dataset. To determine an appropriate cut-off value for a deleterious fitness effect, we calculated the

median Chronos score for each of the 2040 pan-essential genes across the 24 iCCA cell lines. We selected the largest of these median values as our negative fitness threshold (-0.0141220545). Therefore, non-essential genes were identified as enhanced survival dependencies if they met the following criteria: (1) mean Chronos score below the threshold (-0.0141220545) among cell lines in LS-like and/or RP-like subgroups; (2) significant difference in Chronos scores between LS-like and RP-like subgroups (Wilcoxon p<0.05). As true survival dependencies likely arise from multiple genes within common networks, we prioritized enhanced survival dependencies annotated within a common pathway (informed by ENRICHR; described in section 1.6.2). Dependency networks were visualized using CytoScape (StringDB score 0.75) and manually annotated based on MSigDB associations. The potential druggability of genes was investigated using The Drug Gene Interaction Database (DGIdb 4.0) [40].

ANALYSIS OF SINGLE CELL RNA-SEQUENCING DATASETS

Single cell RNA-sequencing dataset pre-processing and filtering

In this manuscript, 3 single cell RNA-sequencing (scRNA-seq) datasets were analysed:

GSE125449 [22]: Data were pre-processed as described in section 1.6.4. Cell type annotation was retained as previously reported by the authors, with the exception of tumour-associated macrophages which were renamed as tumour-associated myeloid cells. Exclusively considering samples where tumour cells were retained, the following cell numbers were included for analysis: 739 tumour cells, 354 cancer-associated fibroblasts, 386 tumour-associated myeloid cells, 2138 T cells (corresponding to 9 samples). A tumour-origin RPLS score (predicted by digital cytometry; section 1.6.4) was calculated for each tumour cell using the 'escape' package. Samples were ranked according to tumour-origin RPLS scores and classified as LS- or RP-like based on median expression.

GSE151530 [41]: Data were pre-processed as described in section 1.6.4. Cell type annotation was retained as previously reported by the authors, with the exception of tumour-associated macrophages which were renamed as tumour-associated myeloid cells. Exclusively considering samples where tumour cells were retained, the following cell numbers were included for analysis: 960 tumour cells, 314 cancer-associated fibroblasts, 478 tumour-associated myeloid cells, 1032 T cells (corresponding to 10 samples). A tumour-origin RPLS score (predicted by digital cytometry; section 1.6.4) was calculated for each tumour cell using

the 'escape' package. Samples were ranked according to tumour-origin RPLS scores and classified as LS- or RP-like based on median expression.

GSE171899 [42]: Data were pre-processed and cells were annotated by the original authors, as indicated in their manuscript. This dataset was exclusively comprised of immune (CD45+) cells and only cells originating from 3 patients who underwent adjuvant chemotherapy were considered: 39 cDC1 (BATF3+), 374 cDC2 (CD1C+), 185 lymphoid, 465 macrophages (ID3+), 365 macrophages (MARCO+), 200 macrophages (TREM2+), 535 monocytes (CD14+), 38 monocytes (FCGR3A). A myeloid-origin RPLS score (predicted by digital cytometry; section 1.6.4) was calculated for each immune cell using the 'escape' package.

Biological analysis of LS-like and RP-like iCCA in scRNA-seq datasets

Biological pathway and process expression were calculated in single cells using the 'escape' package (using MSigDB reference signature databases, as described in section 1.6). The following signatures associated with myeloid cell phenotypes and processes were also used: inflammatory and tolerogenic hepatic macrophages [43]; activated dendritic cells (aDC), immature dendritic cells (iDC), plasmocytoid dendritic cells (pDC) [25]; antigen processing and presenting machinery (APM score) [44]; MHC presentation (classical, class I) [32]; immunoinhibitors and immunostimulators [45]. Transcription factor activities and cytokine activities were also calculated for single cells as earlier described (section 1.6). Ligand:receptor (LR) interactions were identified using CellChat with default settings [46]. Only reproducible interactions (identified in GSE125449 and GSE151530) identified in one tumour subgroup but not the other (LS-like or RP-like) were considered as significant. The potential druggability of genes was investigated using The Drug Gene Interaction Database (DGIdb 4.0) [40]. Clinically actionable targets were identified according to Pharos (Tclin level) [47]. T cell subtypes were assigned using the 'ProjecTILs' package [48]. Immune-oncology targets of interest were defined by the The Cancer Research Institute (CRI) iAtlas consortium (https://isb-cgc.shinyapps.io/shiny-iatlas/) [49]. Metabolic flux inference was performed using METAFLUX[50] with default settings, involving calculation of metabolic reaction activity scores (cubic root normalised) per cell followed by median-based collapsing of activity scores per sample.

O'Rourke CJ, *et al. Gut* 2024; 73:496–508. doi: 10.1136/gutjnl-2023-330748

RESECTED CHOLANGIOCARCINOMA DATASETS

Transcriptome analyses

The following transcriptome datasets were analysed as part of our study:

Andersen cohort: This cohort is comprised of 138 resected iCCA and 37 resected pCCA (with survival data), originating from Australia, Europe (Belgium, France, Germany, Italy), and USA. Transcriptome data (humanRef-8v2 BeadChip and Human HT-12 v3 Expression BeadChip, Illumina) were generated by our group, as previously reported [51, 52]. Data were processed from raw IDAT files using background subtraction (iScan system, Illumina), followed by quantile normalization ('preprocessCore', R package). Data were quantified as normalized signal intensity (arbitrary units, 'a.u.').

Dong cohort: This cohort is comprised of 244 resected iCCA (with survival data), originating from China. Transcriptome data (RNA sequencing) were retrieved from the supplementary information of the original manuscript [53]. Data were quantified using log_2(TPM+1), as reported by the original study authors.

GSE107943 cohort: This cohort is comprised of 30 resected iCCA (with classification as large duct- or small duct-type) from Korea. Transcriptome data (RNA sequencing) were retrieved from GEO (GSE107943) [54, 55]. Data were quantified using RPKM, as reported by the original study authors.

Job cohort: This cohort is comprised of 70 resected iCCA (with survival data), originating from France. Transcriptome data (Human Transcriptome Array 2.0, Affymetrix) were retrieved form ArrayExpress (E-MTAB-6389) [56]. Data were processed from raw CEL files using Robust Multichip Average (RMA) normalization, as implemented in the 'oligo' R package. Data were quantified as normalized signal intensity (arbitrary units, 'a.u.'). Genes with multiple probes were quantified as the median signal intensity across probes.

Jusakul cohort: This cohort is comprised of 81 resected iCCA (with survival data), originating from Asia (Korea, Singapore, Thailand), Brazil and Europe (France, Romania). Transcriptome data (HumanHT-12 V4.0 expression BeadChip) were retrieved from Gene Expression Omnibus (GEO; GSE89747) [57]. Data were extracted from raw IDAT files and underwent quantile normalization ('preprocessCore', R package). Data were quantified as normalized signal intensity (arbitrary units, 'a.u.').

Montal cohort: This cohort is comprised of 182 resected extrahepatic CCA (with survival data), originating from Europe (Spain, Switzerland) and USA. Transcriptome data (Human Genome

U219 Array, Affymetrix) were processed by the original study authors, as reported in the original manuscript [58]. Data were quantified as normalized signal intensity (arbitrary units, 'a.u.').

Nakamura cohort: This cohort is comprised of 111 resected iCCA (with survival data), originating from Japan. Transcriptome data (RNA sequencing) were retrieved from European Genome Phenome Archive (EGA; EGA00001000950) [59] and processed using standard bioinformatic pipelines, as previously reported by us [60]. Data were quantified using log_2(RPKM+1).

Sia cohort: This cohort is comprised of 119 resected iCCA (with survival data), originating from Europe (Italy, Spain) and USA. Transcriptome data (Whole-Genome DASL HT assay, Illumina) were processed by the original study authors, as reported in the original manuscript [61]. Data were quantified as normalized signal intensity (arbitrary units, 'a.u.').

Genomic analyses

Genomic data were available for the Andersen, Dong and Nakamura cohorts, as follows:

Andersen cohort: Mutation data (TruSeq Amplicon Panel, Illumina) were available for 87% (120/138) of this cohort. Data were processed and analyzed as previously reported by our group [60]. *FGFR2* fusion data (quantitative polymerase chain reaction of known breakpoint regions) were also available for 80.4% (111/138) of the cohort [60].

Dong cohort: Mutation data (whole-exome sequencing) and *FGFR2* fusion data (RNA-seq) were available for 97.1% (237/244) and 100% (244/244) of this cohort, as originally reported by the study [53].

Nakamura cohort: Mutation data (whole-exome sequencing) and *FGFR2* fusion data (RNA-seq) were available for 100% (111/111) of this cohort [59], and these data were processed as originally reported by our group [60].

In each of these iCCA cohorts, cancer-relevant hotspot mutations were identified using the Cancer Hotspots database (https://www.cancerhotspots.org/) from Memorial Sloan Kettering Cancer Centre (MSKCC) [62].

ANIMAL MODELS

Changes in RPLS signature expression was evaluated in 14 iCCA-relevant mouse models relative to their study-specific controls. Human homologs were identified for mouse genes

using the 'HMD_HumanPhenotype' file at Mouse Genome Informatics (MGI; informatics.jax.org) [63].

GSE66717: Transcriptome data (Affymetrix Mouse Gene 1.0 ST Array) were downloaded for *Pten-/-* (n=4), *Pten-/- Tgfbr2-/-* (n=4), and control (n=3) mice deposited in GSE66717 [64]. Raw CEL files were subjected to RMA normalization using the 'oligo' R package.

GSE140498: Transcriptome data (Affymetrix Mouse Gene 1.1 ST Array) were downloaded from GSE140498 [65] for the following samples: NEMO single knockout (n=3), NEMO/JNK1Δhepa double knockout (n=3), JNK(1/2)Δhepa double knockout with diethylnitrosamine (DEN) treatment (n=3), JNK(1/2)Δhepa double knockout with bile duct ligation (BDL; n=3), NEMO/JNK(1/2)Δhepa triple knockout (n=3), control mice with DEN treatment (n=3), control mice with bile duct ligation (n=3), and control mice (n=3).

GSE141511: Transcriptome data (RNA-seq) were downloaded for microdissected control (n=3), ductular proliferation (n=3), intraductal papillary neoplasia of the bile duct (IPNB; n=3) and tumour (n=3) tissues from cholangiocyte-directed KrasG12D mice with diet-induced inflammation (3,5-diethoxycarbonyl-1,4-dihydrocollidine (DDC)-treated) deposited in GSE141511 [66]. HTseq count files were merged using 'tximportData' and quantified in counts per million (CPM) using 'edgeR' [67].

AKT/YapS127A: Normalized transcriptome data (Affymetrix array) for this sleeping beauty model of iCCA [68] (n=3) and control mice (n=4) was kindly provided by Prof. Diego Calvisi (Universität Regensburg, Germany).

AKT/NICD: Transcriptome data (Mouse Gene Expression BeadChips, Illumina) for this sleeping beauty model of iCCA [69] (n=4) and control mice (n=3) were generated and analysed as part of our previous study [52].

Genetically engineered models of iCCA: Transcriptome data (RNA-seq) from five iCCA genetically engineered mouse models (GEMMs) harbouring cholangiocyte (CK19)-specific genomic alterations were kindly provided by Dr. Luke Boulter (University of Edinburgh, UK) [70]:, *Kras* & *Nf2* (n=3), *Kras* & *shTrp53* (n=3), *Kras* & *Tp53* (n=3), *Kras* & *Nf2* & *Tp53* (n=3), *Kras* & *Tp53* & *Plbx2* (n=3). Mouse gene IDs were batch annotated using informatics.jax.org.

OTHER CANCER DATASETS

Surgical HCC cohorts

TCGA-LIHC: This cohort is comprised of 359 resected HCC (histologically confirmed and survival data available), originating from USA and published as part of the The Cancer Genome Atlas [71]. Data were generated by RNA-seq and downloaded in RSEM normalized units from Broad GDAC Firehose (https://gdac.broadinstitute.org/). Clinical data were downloaded from cBioPortal (https://www.cbioportal.org/) [72].

GSE14520: This cohort is comprised of 178 resected HCC (histologically confirmed and survival data available). Transcriptome data (Affymetrix Human Genome U133A 2.0 Array) were retrieved from Gene Expression Omnibus (GSE14520) [73]. Data were processed from raw CEL files using Robust Multichip Average (RMA) normalization, as implemented in the 'oligo' R package. Data were quantified as normalized signal intensity (arbitrary units, 'a.u.'). Genes with multiple probes were quantified as the median signal intensity across probes.

Advanced basket cohorts

MET500 cohort: This basket cohort was comprised of 490 metastatic biopsies representative of 22 primary cancer groups [74]. Processed, normalized data (RNA-seq) were downloaded from the following weblink: https://met500.path.med.umich.edu/. Samples were stratified by metastatic sites: abdominal (n=32), liver (n=130), lung & respiratory (n=37), lymph node (n=114), other (n=177).

POG570 cohort: This basket cohort was comprised of 438 metastatic biopsies representative of 26 primary cancer groups [75]. Transcriptome data (RNA sequencing) were downloaded from https://www.bcgsc.ca/downloads/POG570/, followed by Ensembl ID mapping using 'biomaRt'. Data were quantified in TPM. Clinical and treatment information were also downloaded from the POG570 downloads repository: abdominal (n=54), liver (n=198), lung & respiratory (n=37), lymph node (n=87), other (n=62).

Metastatic colorectal cancer cohorts

New EPOC trial: Transcriptome data (normalized Affymetrix array, collapsed by median gene expression) for primary colorectal tumours (n=204) and resected liver metastases (n=145) from the phase III New EPOC trial [76] was kindly provided to us by the S:CORT consortium.

STATISTICAL ANALYSES

Statistical analyses were conducted in R version 4.0.3, unless otherwise stated. Heatmaps were generated using 'gplots'. Bar plots, violin plots, and XY plots were generated in 'ggplot2'. Kaplan-Meier survival analysis and Cox proportional hazards modelling were performed using 'survival' and 'survminer' packages. Forest plots were generated using the 'forestplot' package. Simpson's index was computed using the 'vegan' package. Unrooted dendrograms based on Manhattan distance and complete linkage were generated using 'cluster' and 'ape' packages. Data normality was investigated using Shapiro-Wilk test. Quantitative data were compared across multiple groups using Kruskal-Wallis or ANOVA statistics for non-normal and normal data, respectively. Qualitative data was compared between two groups using Pearson's Chi-squared test, except when one cell in a contingency table has an expected frequency below 5 in which case Fisher's exact test was applied. Correlation analysis was performed using Spearman or Pearson statistics for non-normal and normal data, respectively.

SUPPLEMENTAL FIGURES

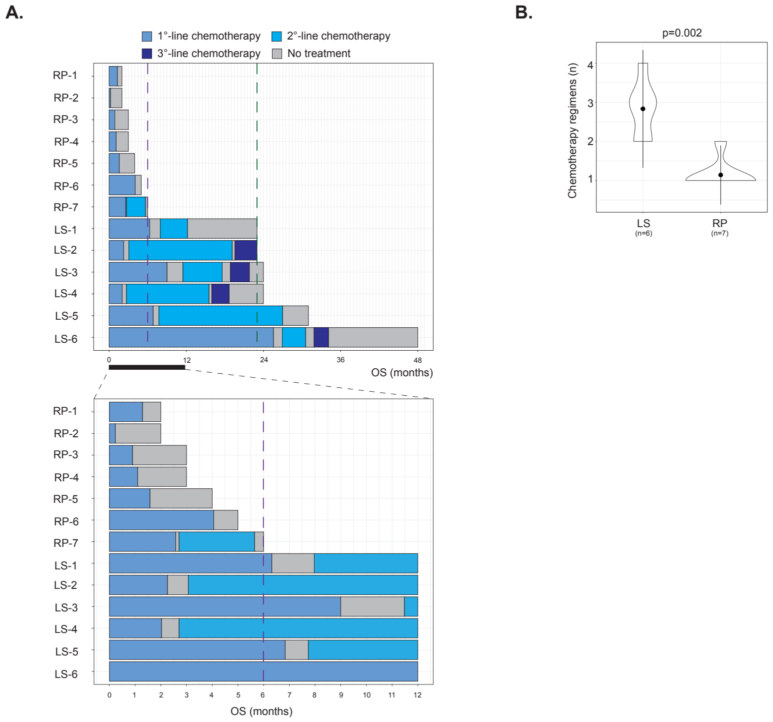

Figure S1: Patient treatment in the RPLS cohort. (A) Swimmer plot of patient management in the RPLS cohort, including a zoomed view on the first 12 months following initiation of

chemotherapy. (B) Comparison of number of chemotherapy regimens (Welch t-test) received by LS (n=6) and RP (n=7) patients.

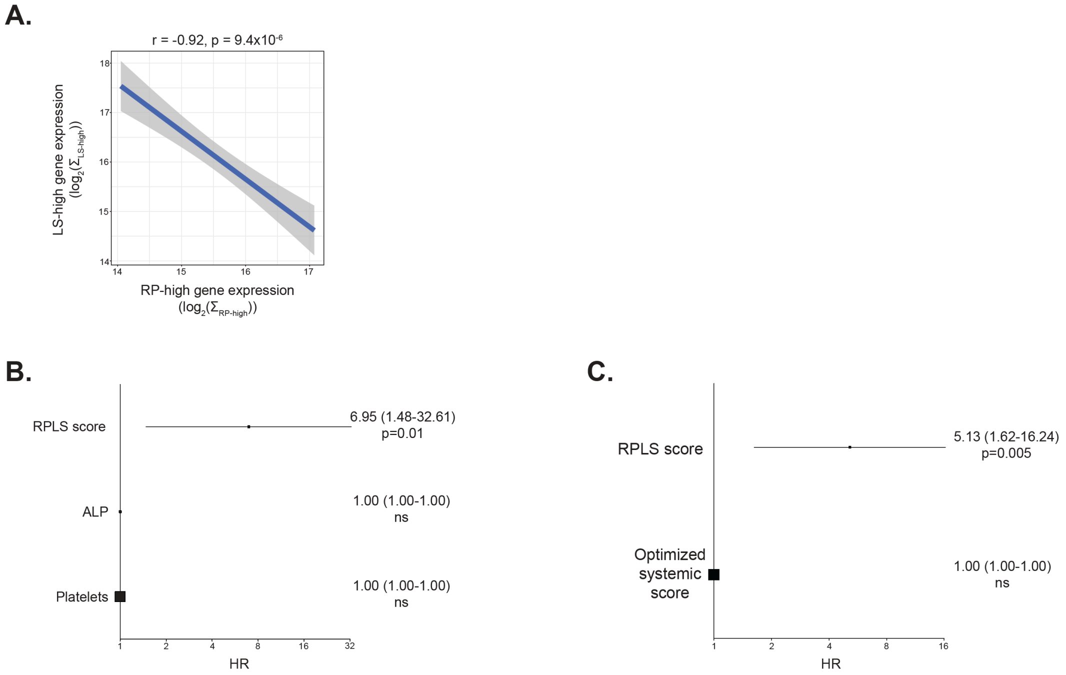

Figure S2: RPLS signature expression and predictive performance in the RPLS cohort. (A) Spearman correlation analysis of RP-high and LS-high gene expression. (B) Forest plot of Cox proportional hazards statistics derived from multivariable analysis of RPLS scores and systemic features differentially expressed between RP and LS patients. HR: hazard ratio; ns: not significant. (C) Forest plot of Cox proportional hazards statistics derived from multivariable analysis of RPLS scores and an optimized systemic signature (defined by AIC backwards elimination using all haematological and systemic features). The final formula derived for this optimized signature was: -211.622 + 41.2*Bili + 1.208*ALP.

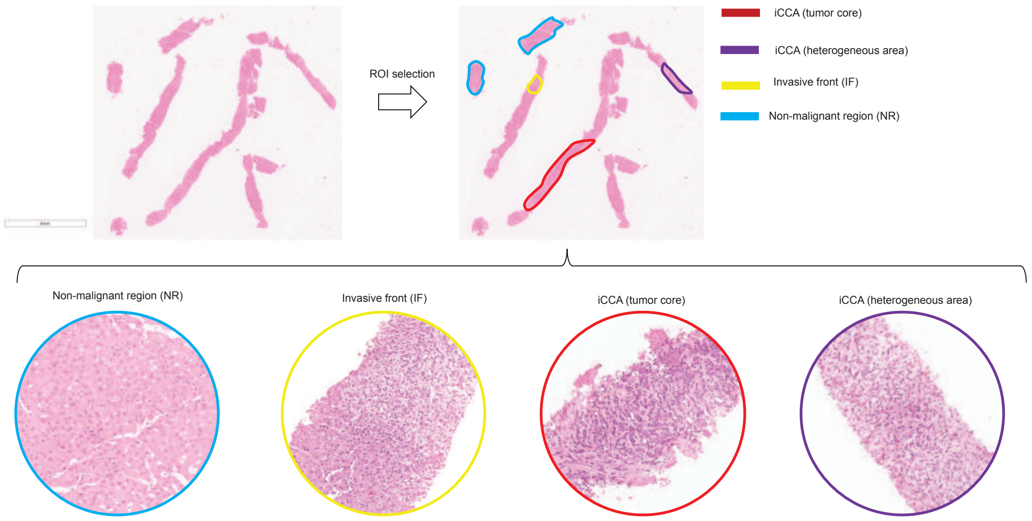

Figure S3: Representative markup of a diagnostic biopsy from the RPLS cohort. Target regions were geospatially macrodissected for whole transcriptome profiling (Tempo-seq).

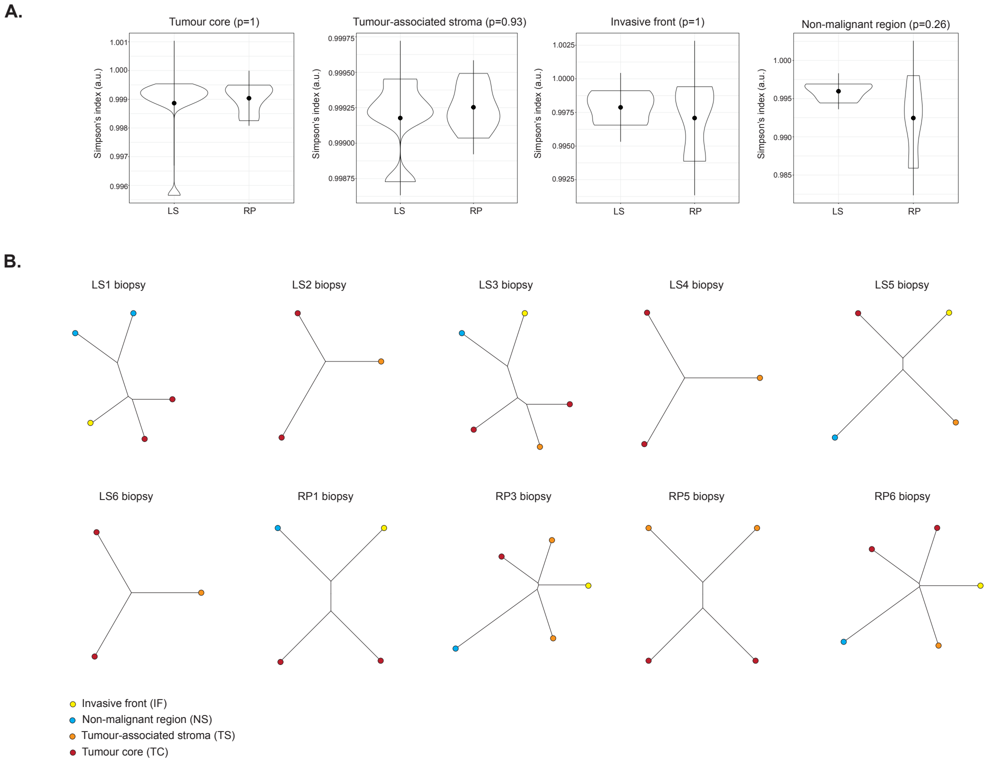

Figure S4: Inter- and intra-sample heterogeneity of macrodissected biopsy regions in the RPLS cohort. (A) Differential intra-sample transcriptomic heterogeneity (Wilcoxon test)

defined by Simpson's index between LS and RP biopsies: tumour cores (TCs; 11 LS, 9 RP), tumour stroma (TSs; 5 LS, 6 RP), invasive fronts (IFs; 3 LS, 3 RP) and non-malignant regions (NRs; 4 LS, 4 RP). (B) Phylotranscriptomic trees (Euclidean distance, Ward linkage) for individual biopsies with 3 or more geospatially macrodissected samples.

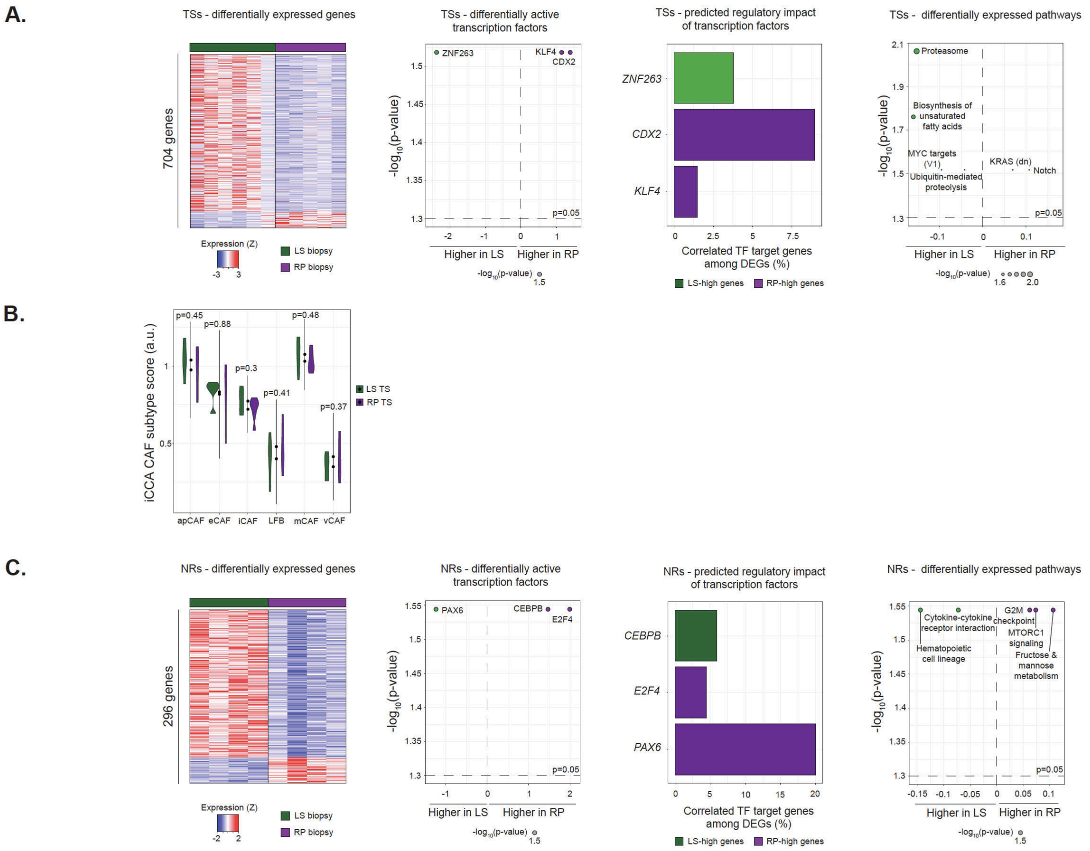

Figure S5: Transcriptomic profiles of tumour stroma and non-malignant regions of biopsies in the RPLS cohort. (A) Differentially expressed genes (min. 2-fold difference, p<0.05, Wilcoxon rank-sum test), differentially active transcription factors (p<0.05, Wilcoxon rank-sum test; DoRothEA), differentially expressed pathways (p<0.05, Wilcoxon rank-sum test; ssGSEA of KEGG and Hallmarks gene lists), and differentially active cytokines (p<0.05, Wilcoxon rank-sum test; CytoSig) between RP (n=6) and LS (n=5) tumour stroma. (B) Differential expression of iCCA cancer-associated fibroblast subtypes in tumour stroma. (C) Differentially expressed genes (min. 2-fold difference, p<0.05, Wilcoxon rank-sum test), differentially active transcription factors (p<0.05, Wilcoxon rank-sum test; DoRothEA), differentially expressed pathways (p<0.05, Wilcoxon rank-sum test; ssGSEA of KEGG and

Hallmarks gene lists), and differentially active cytokines (p<0.05, Wilcoxon rank-sum test; CytoSig) between RP (n=4) and LS (n=4) non-malignant regions.

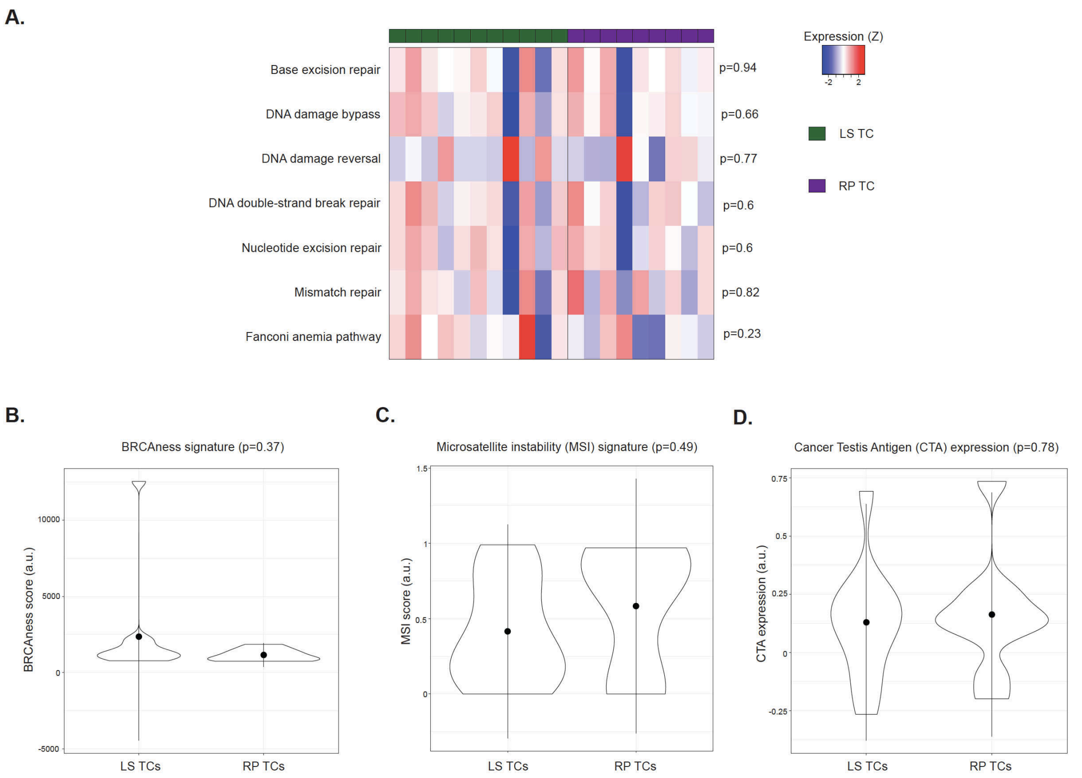

Figure S6: Genomic stability and immunogenicity of tumour cores in the RPLS cohort. Differential expression (Wilcoxon test) of signatures for (A) DNA repair processes (Reactome), (B) BRACness, (C) microsatellite instability, and (D) cancer testis antigen expression in LS and RP biopsies.

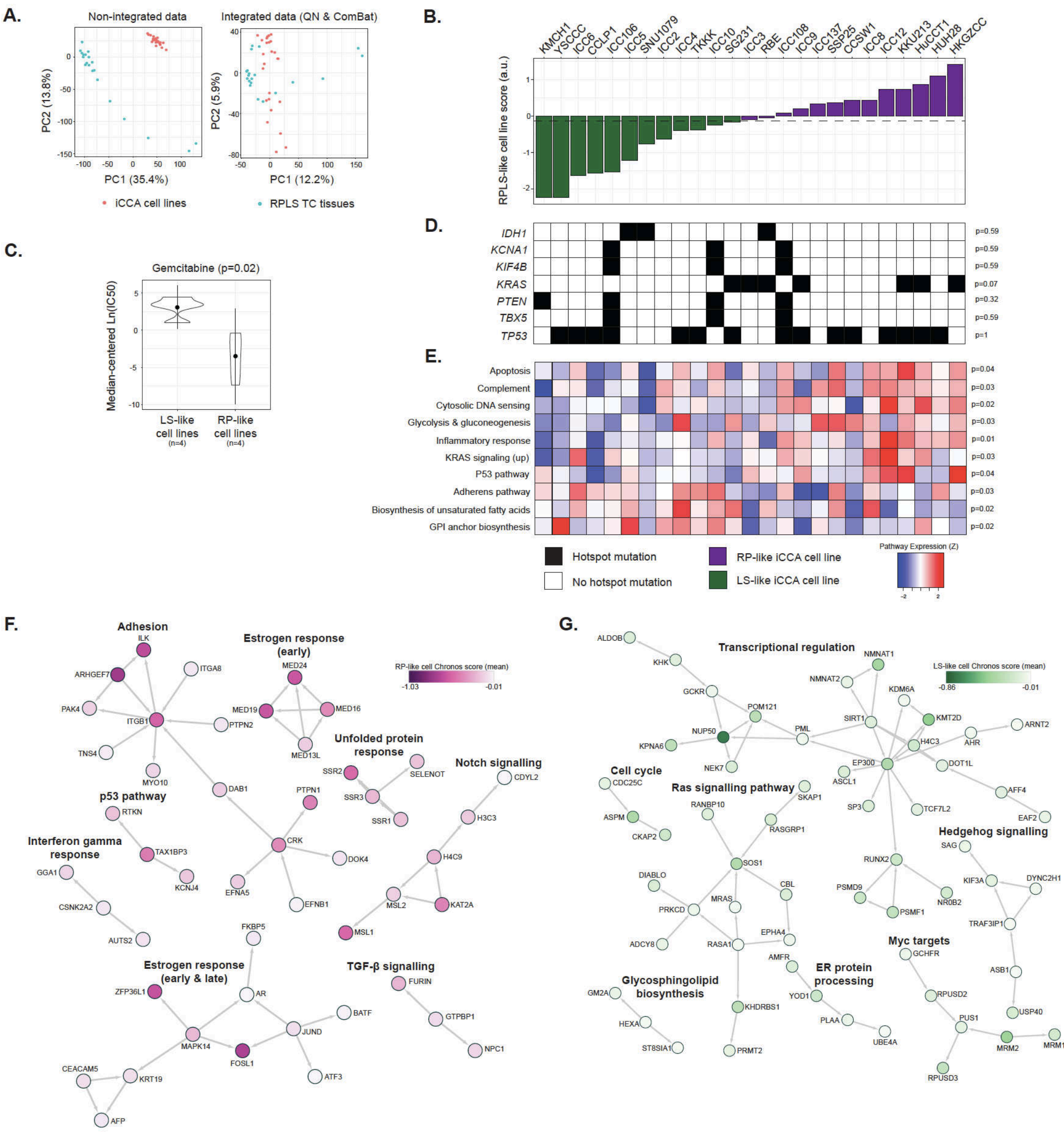

Figure S7: Evaluation of LS- and RP-like iCCA cell lines. (A) Principal component analysis of RPLS tumour core (TC) and iCCA cell line (DepMap) transcriptome profiles before and after data integration. PC: principal component; QN: quantile normalization. (B) Differential sensitivity of LS- and RP-like iCCA cell lines to gemcitabine using data from Saha et al. (Cancer Discovery 2016). IC50: half maximal inhibitory concentration; Ln: natural log. (C) Hotspot mutations in recurrently altered genes across LS- and RP-like cell lines (p-values from fisher's exact test).

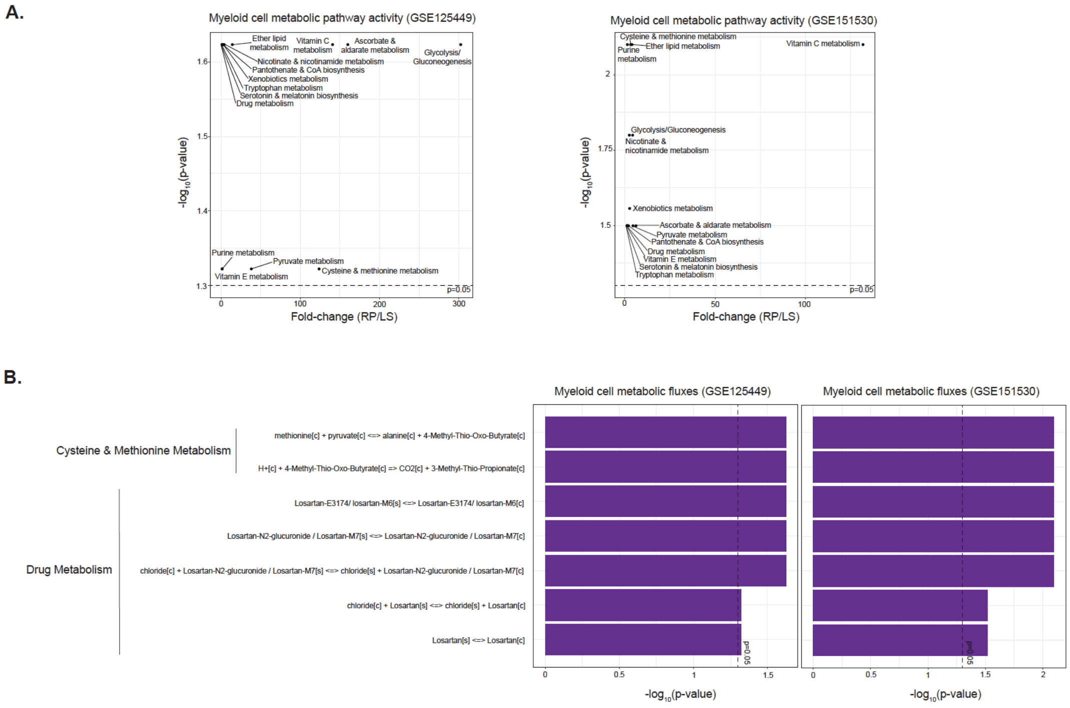

Figure S8: Metabolic flux inference in LS- and RP-like myeloid cells. (A) Differentially active metabolic pathways between myeloid cells in RP-like and LS-like tumours. Activity was inferred using METAFlux in two single cell RNA-sequencing datasets. Only pathways consistently differentially active (Wilcoxon $p<0.05$) in both datasets are shown. (B) Differentially active reactions (within differentially active pathways) between myeloid cells in RP-like and LS-like tumours. Reactions were pre-defined by BiGG Models and activity was inferred using METAflux in two single cell RNA-sequencing datasets. Only reactions consistently differentially active (Wilcoxon $p<0.05$) in both datasets are shown.

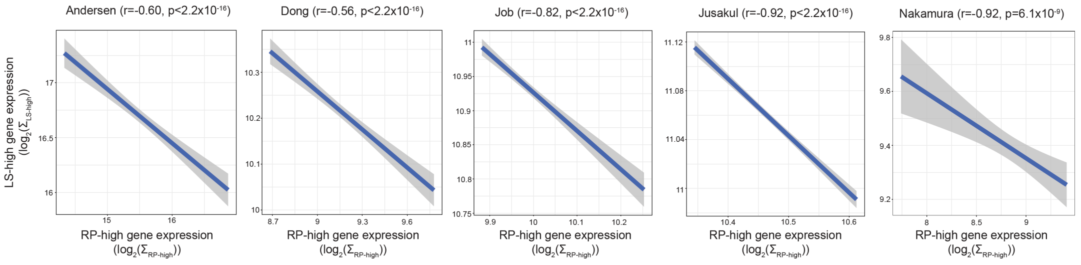

Figure S9: Correlation analysis of RP-high and LS-high gene expression in resected cohorts. Statistics were computed by Spearman's correlation statistics.

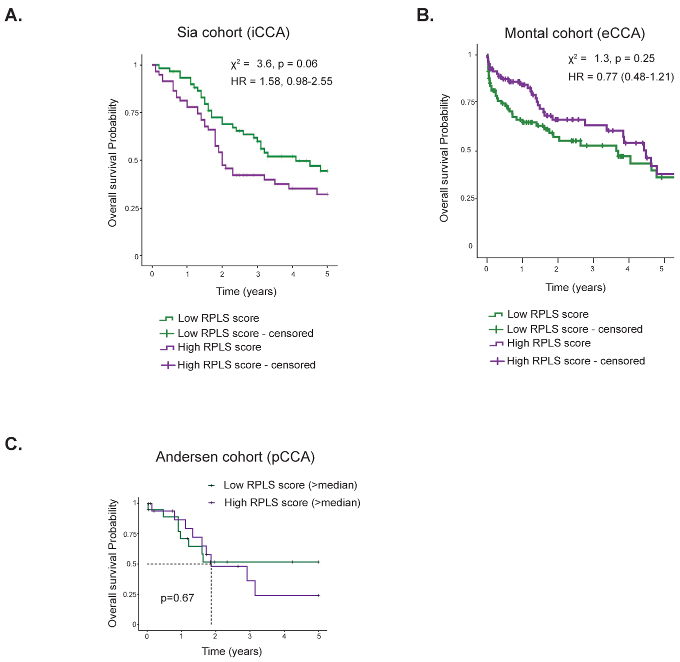

Figure S10: Prognostic associations of RPLS scores in additional resected CCA cohorts. Kaplan-Meier survival curves with log-rank statistics for (A) overall survival in the Sia iCCA cohort (n=149), (B) overall survival in the Montal eCCA cohort (n=182), and (C) overall survival in the Andersen pCCA cohort (n=37). Samples were stratified into high (>median) or low (<median) RPLS score groups.

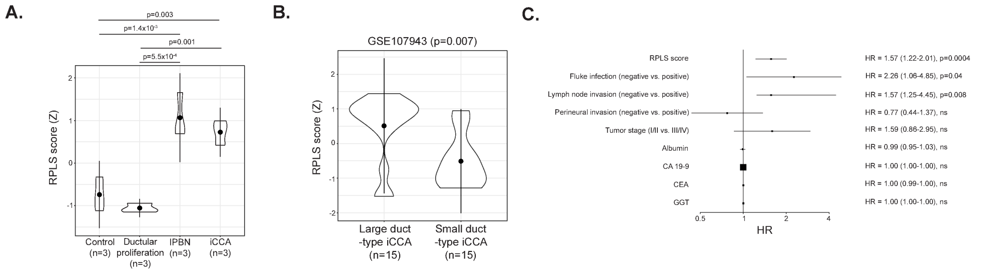

Figure S11: RPLS signature expression in mouse and human iCCA tissues. (A) Differential expression of the RPLS signature in microdissected tissue regions from mice with *Kras*^{G12D} expressing mice and diet-induced cholangitis. P-values were computed by Welch t-test. IPNB: intraductal papillary neoplasm of the bile duct. (B) Differential expression of the RPLS signature between small- (n=15) and large duct-type (n=15) iCCA. P-values were computed by Wilcoxon test. (C) Forest plot of Cox proportional hazards statistics derived from multivariable

analysis of RPLS scores and correlated clinicopathologic variables in the Dong cohort (n=244). γ-GT: γ-glutamyltransferase; CA 19-9: carbohydrate antigen 19-9; CEA: carcinoembryonic antigen; HR: hazard ratio; ns: not significant.

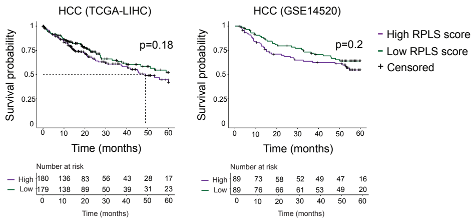

Figure S12: Prognostic associations of the RPLS signature in hepatocellular carcinoma. Kaplan-Meier survival curves with log-rank statistics for high (>median) and low (<median) RPLS signature expression in the TCGA-LIHC and GSE14520 cohorts of resected hepatocellular carcinoma patients.

SUPPLEMENTARY TABLE TITLES

Table S1: Baseline clinical characteristics of the RPLS cohort.

Table S2: Differential expression of the RPLS gene signature.

Table S3: Differential genetic dependencies of RP-like and LS-like iCCA cell lines based on genome-wide CRISPR inactivation screens.

Table S4: Differential pathway expression between RP-like and LS-like tumo**u**r cells in scRNA-seq from iCCA tissues.

Table S5: Differential activity of cytokines and transcription factors between cell types from RP- and LS-like tumo**u**rs.

Table S6: Ligand-receptor interactions specific to RP-like tumo**u**r cells.

Table S7: Druggability of ligand-stimulated receptors on RP-like tumo**u**r cells.

Table S8: Clinicopathologic associations of the RPLS signature across resected iCCA cohorts.

Table S9: Molecular pathobiological differences between liver metastatases and other metastases in advanced cancers.

Table S10: Univariable and multivariable progression-free survival (PFS) analysis of the RPLS signature, clinicopathologic and genomic features in colorectal cancer liver metastases (New EPOC trial).

Table S11: Univariable and multivariable overall survival (OS) analysis of the RPLS signature, clinicopathologic and genomic features in colorectal cancer liver metastases (New EPOC trial).

SUPPLEMENTARY REFERENCES

1 Valle J, Wasan H, Palmer DH, Cunningham D, Anthoney A, Maraveyas A*, et al.* Cisplatin plus gemcitabine versus gemcitabine for biliary tract cancer. N Engl J Med 2010;**362**:1273-81.

2 Bankhead P, Loughrey MB, Fernandez JA, Dombrowski Y, McArt DG, Dunne PD*, et al.* QuPath: Open source software for digital pathology image analysis. Sci Rep 2017;**7**:16878.

3 Love MI, Huber W, Anders S. Moderated estimation of fold change and dispersion for RNA-seq data with DESeq2. Genome Biol 2014;**15**:550.

4 Merritt CR, Ong GT, Church SE, Barker K, Danaher P, Geiss G*, et al.* Multiplex digital spatial profiling of proteins and RNA in fixed tissue. Nat Biotechnol 2020;**38**:586-99.

5 Fisher NC, Byrne RM, Leslie H, Wood C, Legrini A, Cameron AJ*, et al.* Biological Misinterpretation of Transcriptional Signatures in Tumor Samples Can Unknowingly Undermine Mechanistic Understanding and Faithful Alignment with Preclinical Data. Clin Cancer Res 2022;**28**:4056-69.

6 Kanehisa M, Sato Y, Kawashima M, Furumichi M, Tanabe M. KEGG as a reference resource for gene and protein annotation. Nucleic Acids Res 2016;**44**:D457-62.

7 Shannon P, Markiel A, Ozier O, Baliga NS, Wang JT, Ramage D*, et al.* Cytoscape: a software environment for integrated models of biomolecular interaction networks. Genome Res 2003;**13**:2498-504.

8 Merico D, Isserlin R, Stueker O, Emili A, Bader GD. Enrichment map: a network-based method for gene-set enrichment visualization and interpretation. PLoS One 2010;**5**:e13984.

9 Kucera M, Isserlin R, Arkhangorodsky A, Bader GD. AutoAnnotate: A Cytoscape app for summarizing networks with semantic annotations. F1000Res 2016;**5**:1717.

10 Hanzelmann S, Castelo R, Guinney J. GSVA: gene set variation analysis for microarray and RNA-seq data. BMC Bioinformatics 2013;**14**:7.

11 Liberzon A, Birger C, Thorvaldsdottir H, Ghandi M, Mesirov JP, Tamayo P. The Molecular Signatures Database (MSigDB) hallmark gene set collection. Cell Syst 2015;**1**:417-25.

12 Subramanian A, Tamayo P, Mootha VK, Mukherjee S, Ebert BL, Gillette MA*, et al.* Gene set enrichment analysis: a knowledge-based approach for interpreting genome-wide expression profiles. Proc Natl Acad Sci U S A 2005;**102**:15545-50.

13 Liberzon A, Subramanian A, Pinchback R, Thorvaldsdottir H, Tamayo P, Mesirov JP. Molecular signatures database (MSigDB) 3.0. Bioinformatics 2011;**27**:1739-40.

14 Garcia-Alonso L, Holland CH, Ibrahim MM, Turei D, Saez-Rodriguez J. Benchmark and integration of resources for the estimation of human transcription factor activities. Genome Res 2019;**29**:1363-75.

15 Nakano Y, Tanno S, Koizumi K, Nishikawa T, Nakamura K, Minoguchi M*, et al.* Gemcitabine chemoresistance and molecular markers associated with gemcitabine transport and metabolism in human pancreatic cancer cells. Br J Cancer 2007;**96**:457-63.
16 Sebastiani V, Ricci F, Rubio-Viqueira B, Kulesza P, Yeo CJ, Hidalgo M*, et al.* Immunohistochemical and genetic evaluation of deoxycytidine kinase in pancreatic cancer: relationship to molecular mechanisms of gemcitabine resistance and survival. Clin Cancer Res 2006;**12**:2492-7.
17 Vos LJ, Yusuf D, Lui A, Abdelaziz Z, Ghosh S, Spratlin JL*, et al.* Predictive and Prognostic Properties of Human Equilibrative Nucleoside Transporter 1 Expression in Gemcitabine-Treated Pancreatobiliary Cancer: A Meta-Analysis. JCO Precis Oncol 2019;**3**:1-22.
18 Mourragui SMC, Loog M, Vis DJ, Moore K, Manjon AG, van de Wiel MA*, et al.* Predicting patient response with models trained on cell lines and patient-derived xenografts by nonlinear transfer learning. Proc Natl Acad Sci U S A 2021;**118**.
19 Nicolle R, Gayet O, Duconseil P, Vanbrugghe C, Roques J, Bigonnet M*, et al.* A transcriptomic signature to predict adjuvant gemcitabine sensitivity in pancreatic adenocarcinoma. Ann Oncol 2021;**32**:250-60.
20 Tiriac H, Belleau P, Engle DD, Plenker D, Deschenes A, Somerville TDD*, et al.* Organoid Profiling Identifies Common Responders to Chemotherapy in Pancreatic Cancer. Cancer Discov 2018;**8**:1112-29.
21 Newman AM, Steen CB, Liu CL, Gentles AJ, Chaudhuri AA, Scherer F*, et al.* Determining cell type abundance and expression from bulk tissues with digital cytometry. Nat Biotechnol 2019;**37**:773-82.
22 Ma L, Hernandez MO, Zhao Y, Mehta M, Tran B, Kelly M*, et al.* Tumor Cell Biodiversity Drives Microenvironmental Reprogramming in Liver Cancer. Cancer Cell 2019;**36**:418-30 e6.
23 Ma L, Wang L, Khatib SA, Chang CW, Heinrich S, Dominguez DA*, et al.* Single-cell atlas of tumor cell evolution in response to therapy in hepatocellular carcinoma and intrahepatic cholangiocarcinoma. J Hepatol 2021.
24 Stuart T, Butler A, Hoffman P, Hafemeister C, Papalexi E, Mauck WM, 3rd*, et al.* Comprehensive Integration of Single-Cell Data. Cell 2019;**177**:1888-902 e21.
25 Aran D, Hu Z, Butte AJ. xCell: digitally portraying the tissue cellular heterogeneity landscape. Genome Biol 2017;**18**:220.
26 Sturm G, Finotello F, List M. Immunedeconv: An R Package for Unified Access to Computational Methods for Estimating Immune Cell Fractions from Bulk RNA-Sequencing Data. Methods Mol Biol 2020;**2120**:223-32.
27 Rooney MS, Shukla SA, Wu CJ, Getz G, Hacohen N. Molecular and genetic properties of tumors associated with local immune cytolytic activity. Cell 2015;**160**:48-61.
28 Jiang P, Zhang Y, Ru B, Yang Y, Vu T, Paul R*, et al.* Systematic investigation of cytokine signaling activity at the tissue and single-cell levels. Nat Methods 2021;**18**:1181-91.
29 Almeida LG, Sakabe NJ, deOliveira AR, Silva MC, Mundstein AS, Cohen T*, et al.* CTdatabase: a knowledge-base of high-throughput and curated data on cancer-testis antigens. Nucleic Acids Res 2009;**37**:D816-9.
30 Jiang P, Gu S, Pan D, Fu J, Sahu A, Hu X*, et al.* Signatures of T cell dysfunction and exclusion predict cancer immunotherapy response. Nat Med 2018;**24**:1550-8.
31 Konstantinopoulos PA, Spentzos D, Karlan BY, Taniguchi T, Fountzilas E, Francoeur N*, et al.* Gene expression profile of BRCAness that correlates with responsiveness to

chemotherapy and with outcome in patients with epithelial ovarian cancer. J Clin Oncol 2010;**28**:3555-61.
32 Gillespie M, Jassal B, Stephan R, Milacic M, Rothfels K, Senff-Ribeiro A*, et al.* The reactome pathway knowledgebase 2022. Nucleic Acids Res 2022;**50**:D687-D92.
33 Affo S, Nair A, Brundu F, Ravichandra A, Bhattacharjee S, Matsuda M*, et al.* Promotion of cholangiocarcinoma growth by diverse cancer-associated fibroblast subpopulations. Cancer Cell 2021;**39**:866-82 e11.
34 Ghandi M, Huang FW, Jane-Valbuena J, Kryukov GV, Lo CC, McDonald ER, 3rd*, et al.* Next-generation characterization of the Cancer Cell Line Encyclopedia. Nature 2019;**569**:503-8.
35 Leek JT, Johnson WE, Parker HS, Jaffe AE, Storey JD. The sva package for removing batch effects and other unwanted variation in high-throughput experiments. Bioinformatics 2012;**28**:882-3.
36 Saha SK, Gordan JD, Kleinstiver BP, Vu P, Najem MS, Yeo JC*, et al.* Isocitrate Dehydrogenase Mutations Confer Dasatinib Hypersensitivity and SRC Dependence in Intrahepatic Cholangiocarcinoma. Cancer Discov 2016;**6**:727-39.
37 Pacini C, Dempster JM, Boyle I, Goncalves E, Najgebauer H, Karakoc E*, et al.* Integrated cross-study datasets of genetic dependencies in cancer. Nat Commun 2021;**12**:1661.
38 Dempster JM, Boyle I, Vazquez F, Root DE, Boehm JS, Hahn WC*, et al.* Chronos: a cell population dynamics model of CRISPR experiments that improves inference of gene fitness effects. Genome Biol 2021;**22**:343.
39 Dempster JM, Pacini C, Pantel S, Behan FM, Green T, Krill-Burger J*, et al.* Agreement between two large pan-cancer CRISPR-Cas9 gene dependency data sets. Nat Commun 2019;**10**:5817.
40 Freshour SL, Kiwala S, Cotto KC, Coffman AC, McMichael JF, Song JJ*, et al.* Integration of the Drug-Gene Interaction Database (DGIdb 4.0) with open crowdsource efforts. Nucleic Acids Res 2021;**49**:D1144-D51.
41 Ma L, Wang L, Khatib SA, Chang CW, Heinrich S, Dominguez DA*, et al.* Single-cell atlas of tumor cell evolution in response to therapy in hepatocellular carcinoma and intrahepatic cholangiocarcinoma. J Hepatol 2021;**75**:1397-408.
42 Alvisi G, Termanini A, Soldani C, Portale F, Carriero R, Pilipow K*, et al.* Multimodal single-cell profiling of intrahepatic cholangiocarcinoma defines hyperactivated Tregs as a potential therapeutic target. J Hepatol 2022;**77**:1359-72.
43 MacParland SA, Liu JC, Ma XZ, Innes BT, Bartczak AM, Gage BK*, et al.* Single cell RNA sequencing of human liver reveals distinct intrahepatic macrophage populations. Nat Commun 2018;**9**:4383.
44 Wang S, He Z, Wang X, Li H, Liu XS. Antigen presentation and tumor immunogenicity in cancer immunotherapy response prediction. Elife 2019;**8**.
45 Charoentong P, Finotello F, Angelova M, Mayer C, Efremova M, Rieder D*, et al.* Pan-cancer Immunogenomic Analyses Reveal Genotype-Immunophenotype Relationships and Predictors of Response to Checkpoint Blockade. Cell Rep 2017;**18**:248-62.
46 Jin S, Guerrero-Juarez CF, Zhang L, Chang I, Ramos R, Kuan CH*, et al.* Inference and analysis of cell-cell communication using CellChat. Nat Commun 2021;**12**:1088.
47 Sheils TK, Mathias SL, Kelleher KJ, Siramshetty VB, Nguyen DT, Bologa CG*, et al.* TCRD and Pharos 2021: mining the human proteome for disease biology. Nucleic Acids Res 2021;**49**:D1334-D46.

48 Andreatta M, Corria-Osorio J, Muller S, Cubas R, Coukos G, Carmona SJ. Interpretation of T cell states from single-cell transcriptomics data using reference atlases. Nat Commun 2021;**12**:2965.
49 Eddy JA, Thorsson V, Lamb AE, Gibbs DL, Heimann C, Yu JX*, et al.* CRI iAtlas: an interactive portal for immuno-oncology research. F1000Res 2020;**9**:1028.
50 Huang Y, Mohanty V, Dede M, Tsai K, Daher M, Li L*, et al.* Characterizing cancer metabolism from bulk and single-cell RNA-seq data using METAFlux. Nat Commun 2023;**14**:4883.
51 Andersen JB, Spee B, Blechacz BR, Avital I, Komuta M, Barbour A*, et al.* Genomic and genetic characterization of cholangiocarcinoma identifies therapeutic targets for tyrosine kinase inhibitors. Gastroenterology 2012;**142**:1021-31 e15.
52 O'Rourke CJ, Matter MS, Nepal C, Caetano-Oliveira R, Ton PT, Factor VM*, et al.* Identification of a Pan-Gamma-Secretase Inhibitor Response Signature for Notch-Driven Cholangiocarcinoma. Hepatology 2020;**71**:196-213.
53 Dong L, Lu D, Chen R, Lin Y, Zhu H, Zhang Z*, et al.* Proteogenomic characterization identifies clinically relevant subgroups of intrahepatic cholangiocarcinoma. Cancer Cell 2022;**40**:70-87 e15.
54 Ahn KS, O'Brien D, Kang YN, Mounajjed T, Kim YH, Kim TS*, et al.* Prognostic subclass of intrahepatic cholangiocarcinoma by integrative molecular-clinical analysis and potential targeted approach. Hepatol Int 2019;**13**:490-500.
55 Ahn KS, Kang KJ, Kim YH, Kim TS, Song BI, Kim HW*, et al.* Genetic features associated with (18)F-FDG uptake in intrahepatic cholangiocarcinoma. Ann Surg Treat Res 2019;**96**:153-61.
56 Job S, Rapoud D, Dos Santos A, Gonzalez P, Desterke C, Pascal G*, et al.* Identification of Four Immune Subtypes Characterized by Distinct Composition and Functions of Tumor Microenvironment in Intrahepatic Cholangiocarcinoma. Hepatology 2020;**72**:965-81.
57 Jusakul A, Cutcutache I, Yong CH, Lim JQ, Huang MN, Padmanabhan N*, et al.* Whole-Genome and Epigenomic Landscapes of Etiologically Distinct Subtypes of Cholangiocarcinoma. Cancer Discov 2017;**7**:1116-35.
58 Montal R, Sia D, Montironi C, Leow WQ, Esteban-Fabro R, Pinyol R*, et al.* Molecular classification and therapeutic targets in extrahepatic cholangiocarcinoma. J Hepatol 2020;**73**:315-27.
59 Nakamura H, Arai Y, Totoki Y, Shirota T, Elzawahry A, Kato M*, et al.* Genomic spectra of biliary tract cancer. Nat Genet 2015;**47**:1003-10.
60 Nepal C, O'Rourke CJ, Oliveira D, Taranta A, Shema S, Gautam P*, et al.* Genomic perturbations reveal distinct regulatory networks in intrahepatic cholangiocarcinoma. Hepatology 2018;**68**:949-63.
61 Sia D, Hoshida Y, Villanueva A, Roayaie S, Ferrer J, Tabak B*, et al.* Integrative molecular analysis of intrahepatic cholangiocarcinoma reveals 2 classes that have different outcomes. Gastroenterology 2013;**144**:829-40.
62 Chang MT, Bhattarai TS, Schram AM, Bielski CM, Donoghue MTA, Jonsson P*, et al.* Accelerating Discovery of Functional Mutant Alleles in Cancer. Cancer Discov 2018;**8**:174-83.
63 Bult CJ, Blake JA, Smith CL, Kadin JA, Richardson JE, Mouse Genome Database G. Mouse Genome Database (MGD) 2019. Nucleic Acids Res 2019;**47**:D801-D6.
64 Mu X, Pradere JP, Affo S, Dapito DH, Friedman R, Lefkovitch JH*, et al.* Epithelial Transforming Growth Factor-beta Signaling Does Not Contribute to Liver Fibrosis but Protects Mice From Cholangiocarcinoma. Gastroenterology 2016;**150**:720-33.

65 Cubero FJ, Mohamed MR, Woitok MM, Zhao G, Hatting M, Nevzorova YA*, et al.* Loss of c-Jun N-terminal Kinase 1 and 2 Function in Liver Epithelial Cells Triggers Biliary Hyperproliferation Resembling Cholangiocarcinoma. Hepatol Commun 2020;**4**:834-51.
66 Di-Luoffo M, Pirenne S, Saandi T, Loriot A, Gerard C, Dauguet N*, et al.* A novel mouse model of cholangiocarcinoma uncovers a role for Tensin-4 in tumor progression. Hepatology 2021.
67 Robinson MD, McCarthy DJ, Smyth GK. edgeR: a Bioconductor package for differential expression analysis of digital gene expression data. Bioinformatics 2010;**26**:139-40.
68 Zhang S, Song X, Cao D, Xu Z, Fan B, Che L*, et al.* Pan-mTOR inhibitor MLN0128 is effective against intrahepatic cholangiocarcinoma in mice. J Hepatol 2017;**67**:1194-203.
69 Fan B, Malato Y, Calvisi DF, Naqvi S, Razumilava N, Ribback S*, et al.* Cholangiocarcinomas can originate from hepatocytes in mice. J Clin Invest 2012;**122**:2911-5.
70 Younger NT, Wilson ML, Martinez Lyons A, Jarman EJ, Meynert AM, Grimes GR*, et al.* In Vivo Modeling of Patient Genetic Heterogeneity Identifies New Ways to Target Cholangiocarcinoma. Cancer Res 2022;**82**:1548-59.
71 Cancer Genome Atlas Research Network. Electronic address wbe, Cancer Genome Atlas Research N. Comprehensive and Integrative Genomic Characterization of Hepatocellular Carcinoma. Cell 2017;**169**:1327-41 e23.
72 Cerami E, Gao J, Dogrusoz U, Gross BE, Sumer SO, Aksoy BA*, et al.* The cBio cancer genomics portal: an open platform for exploring multidimensional cancer genomics data. Cancer Discov 2012;**2**:401-4.
73 Roessler S, Jia HL, Budhu A, Forgues M, Ye QH, Lee JS*, et al.* A unique metastasis gene signature enables prediction of tumor relapse in early-stage hepatocellular carcinoma patients. Cancer Res 2010;**70**:10202-12.
74 Robinson DR, Wu YM, Lonigro RJ, Vats P, Cobain E, Everett J*, et al.* Integrative clinical genomics of metastatic cancer. Nature 2017;**548**:297-303.
75 Pleasance E, Bohm A, Williamson LM, Nelson JMT, Shen Y, Bonakdar M*, et al.* Whole genome and transcriptome analysis enhances precision cancer treatment options. Ann Oncol 2022.
76 Bridgewater JA, Pugh SA, Maishman T, Eminton Z, Mellor J, Whitehead A*, et al.* Systemic chemotherapy with or without cetuximab in patients with resectable colorectal liver metastasis (New EPOC): long-term results of a multicentre, randomised, controlled, phase 3 trial. Lancet Oncol 2020;**21**:398-411.
