# Supplementary figures and images for: Molecular portraits of patients with intrahepatic cholangiocarcinoma who diverge as rapid progressors or long survivors on chemotherapy
Source: Gut. 2023 Sep 27;73(3):496–508. doi: 10.1136/gutjnl-2023-330748 (PMC10894814; doi:10.1136/gutjnl-2023-330748)

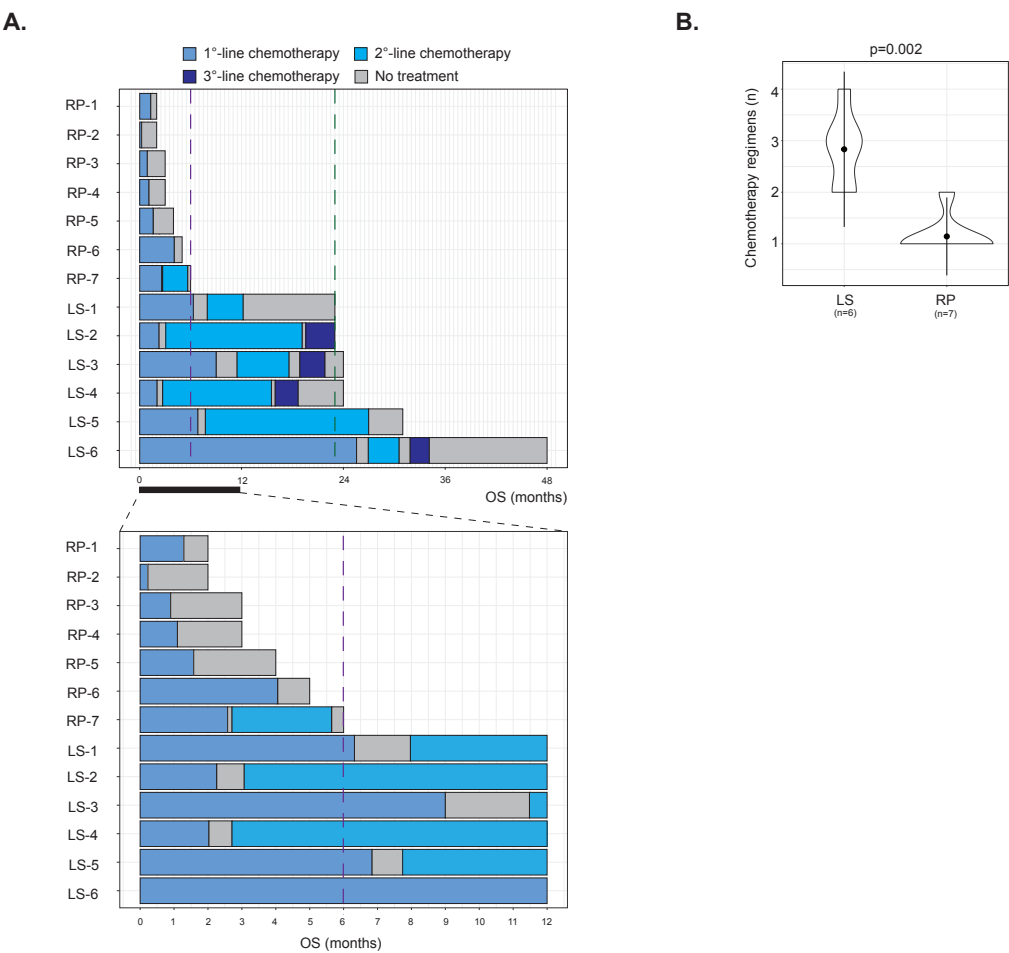

FIGURE S1

Supplement: Supplementary data [file gutjnl-2023-330748supp003.pdf]

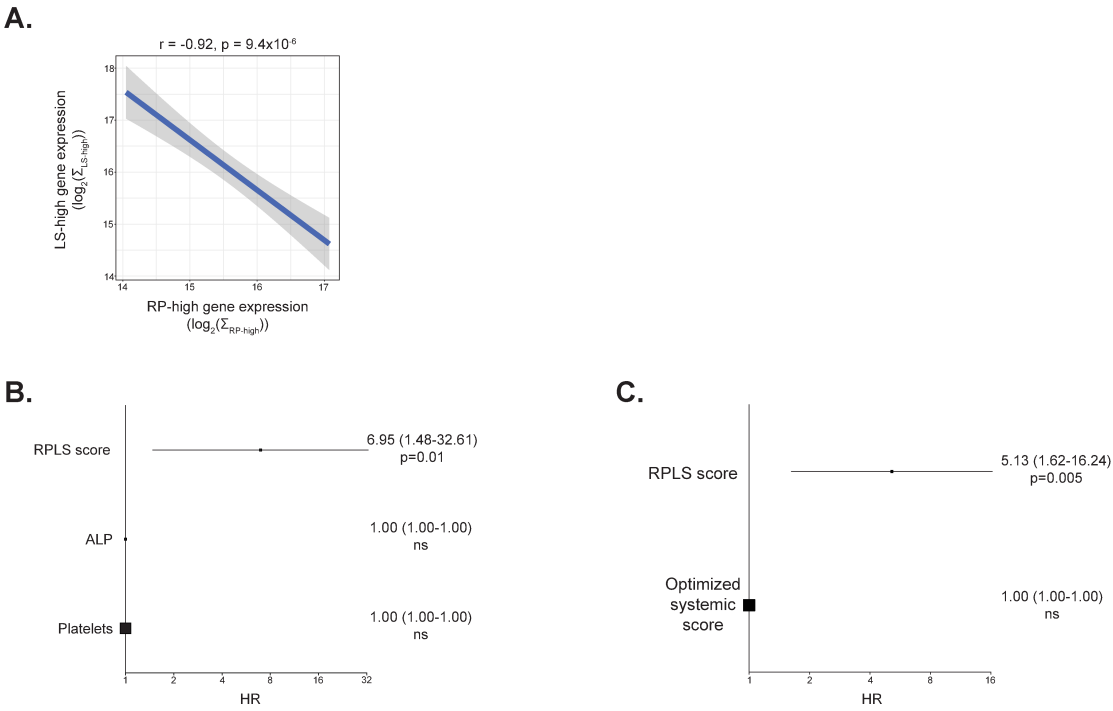

FIGURE S2

Supplement: Supplementary data [file gutjnl-2023-330748supp004.pdf]

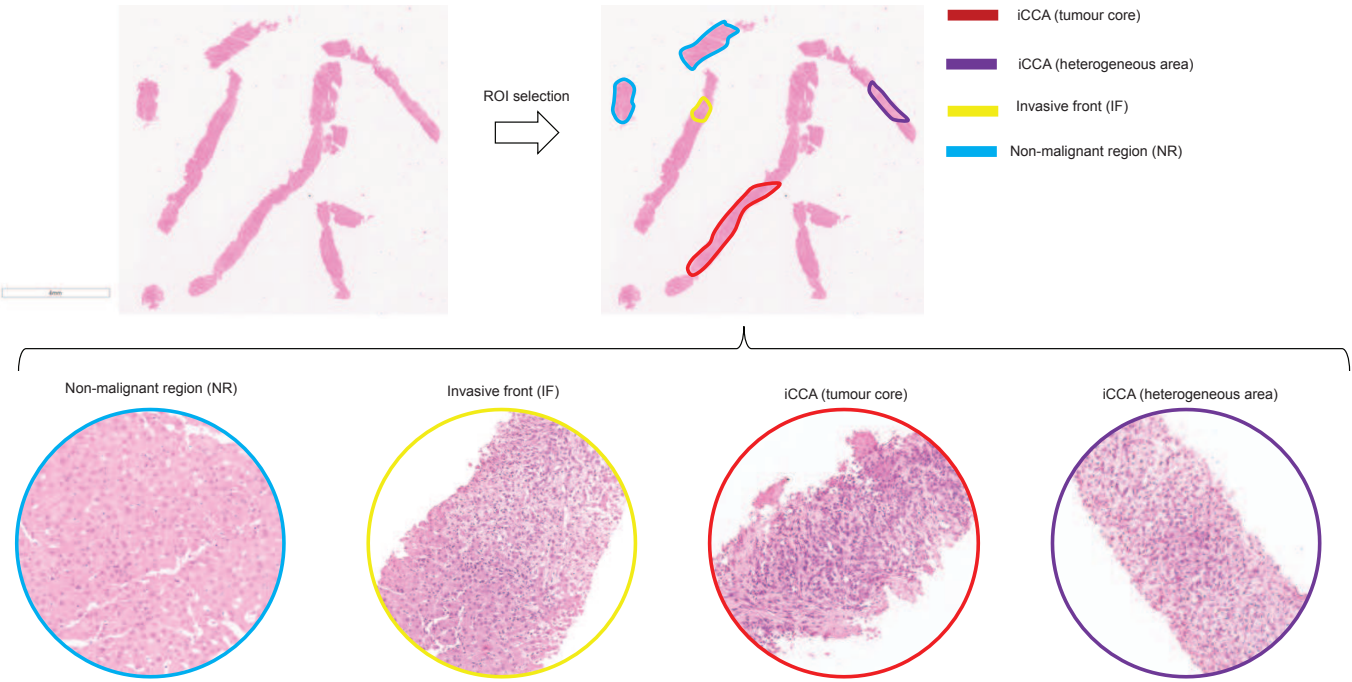

FIGURE S3

Supplement: Supplementary data [file gutjnl-2023-330748supp005.pdf]

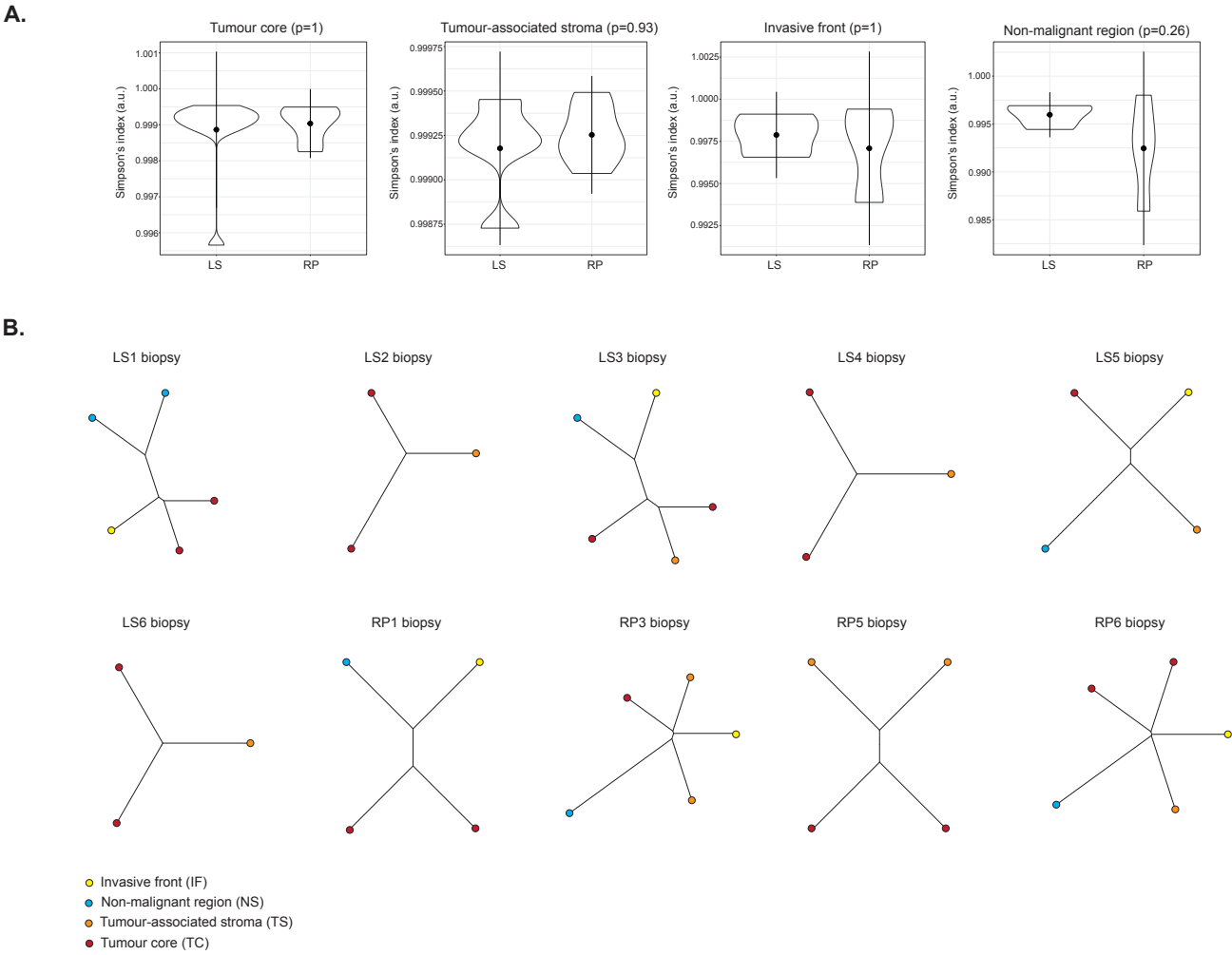

FIGURE S4

Supplement: Supplementary data [file gutjnl-2023-330748supp006.pdf]

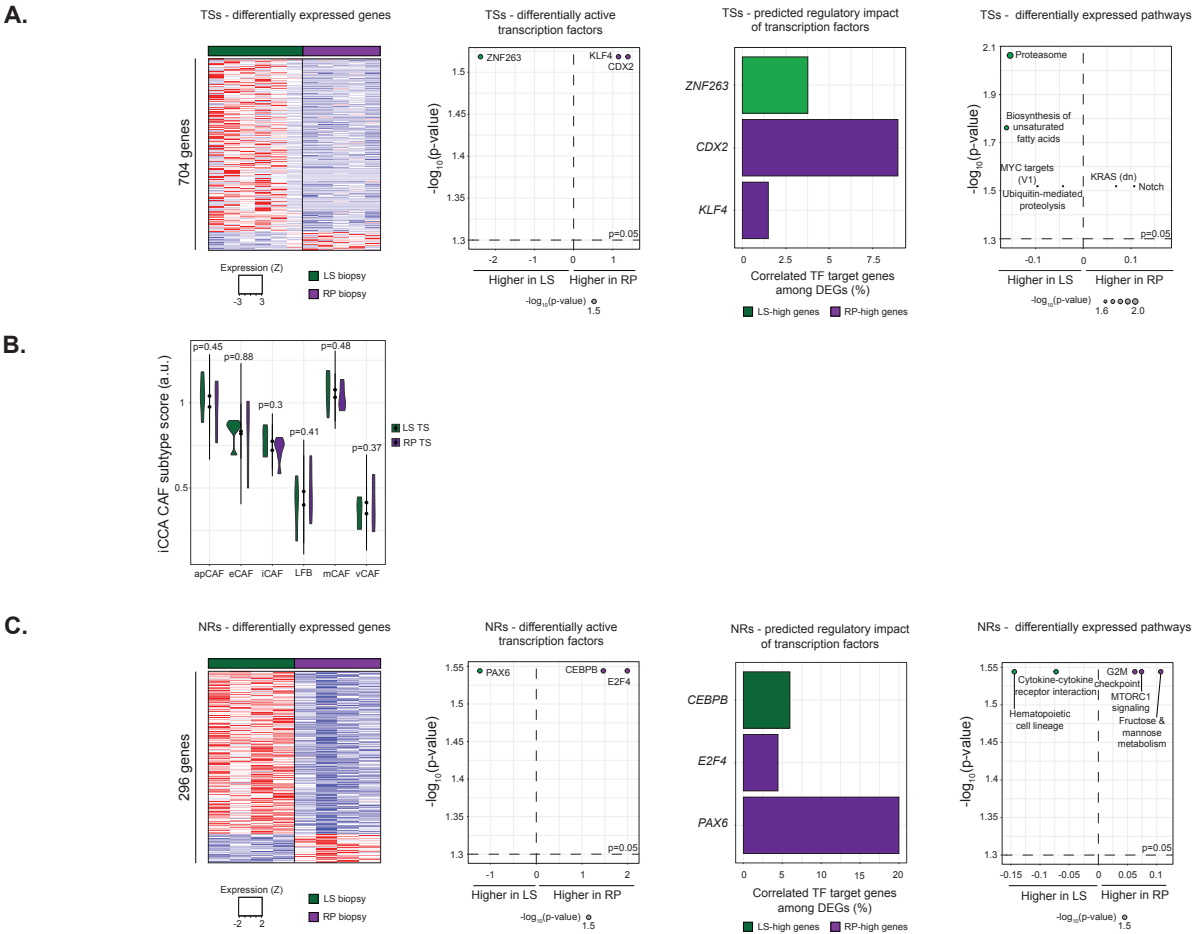

FIGURE S5

Supplement: Supplementary data [file gutjnl-2023-330748supp007.pdf]

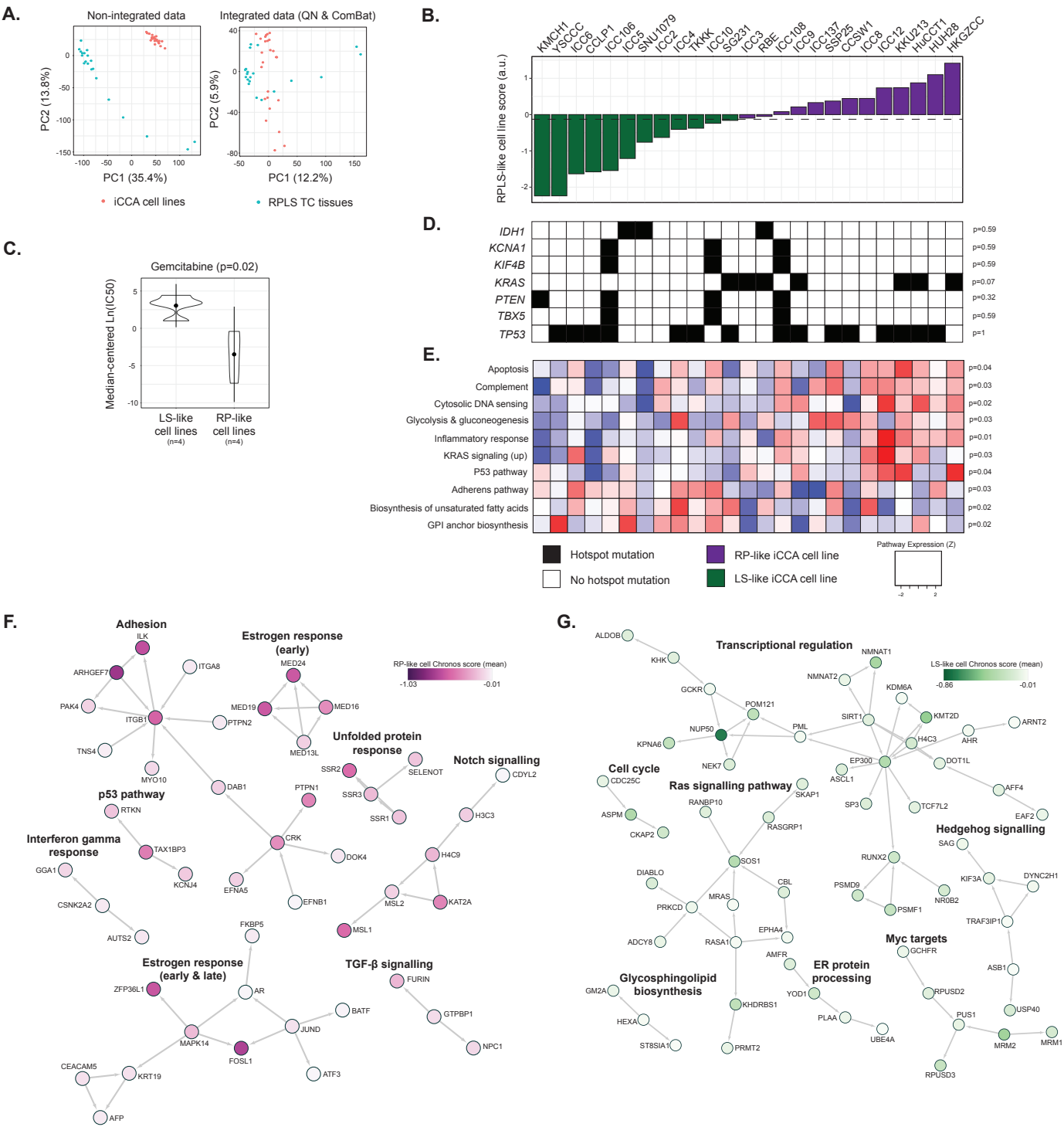

FIGURE S7

Supplement: Supplementary data [file gutjnl-2023-330748supp009.pdf]

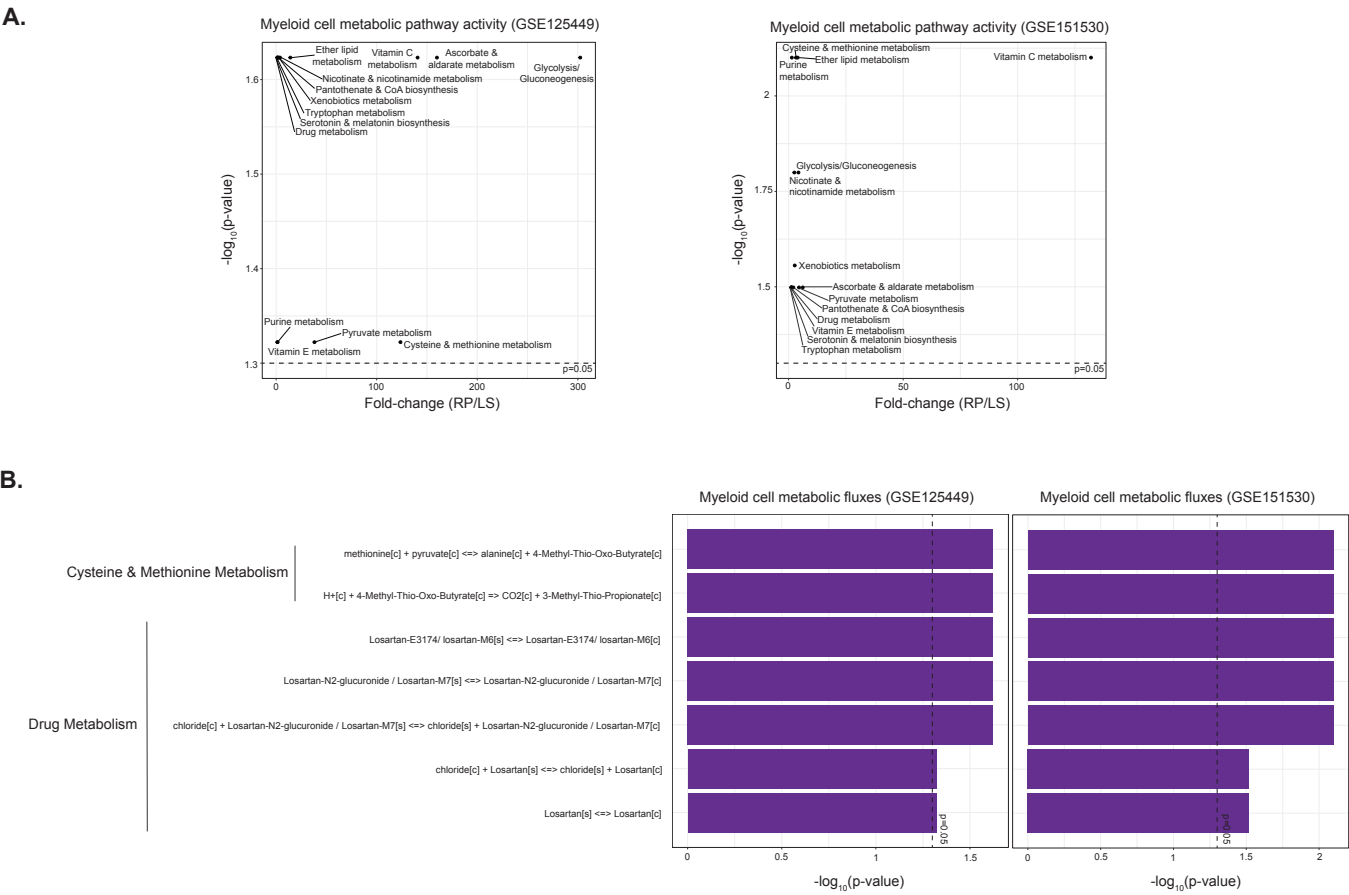

FIGURE S8

Supplement: Supplementary data [file gutjnl-2023-330748supp010.pdf]

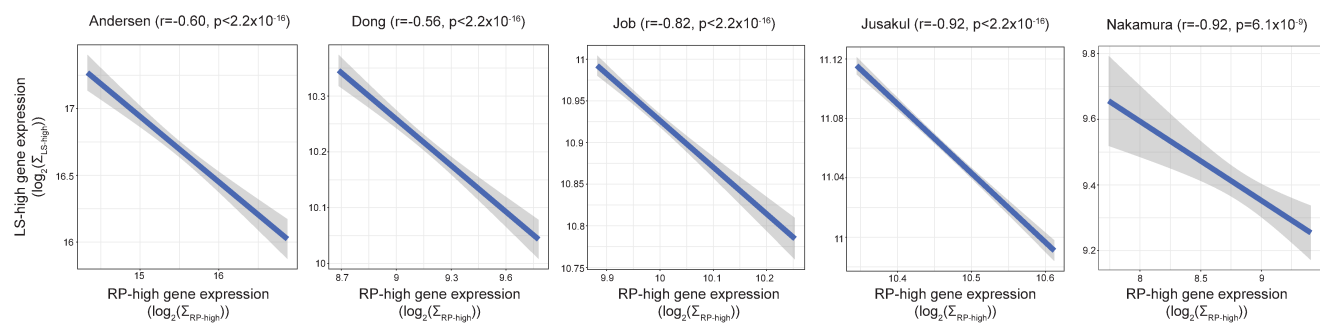

FIGURE S9

Supplement: Supplementary data [file gutjnl-2023-330748supp011.pdf]

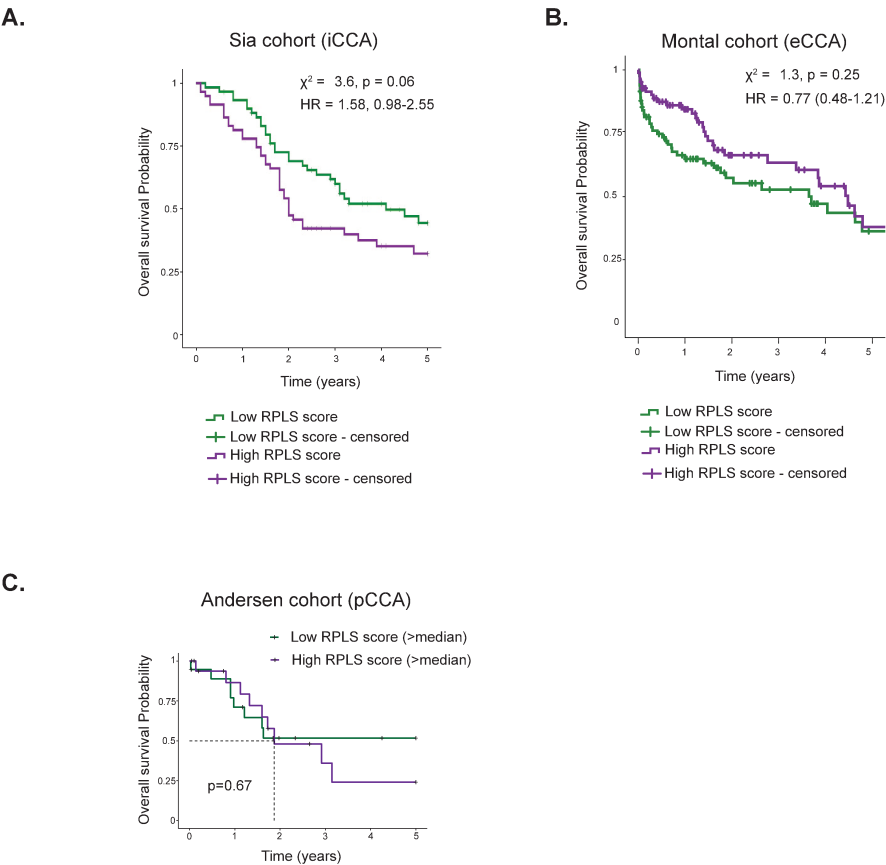

Supplement: Supplementary data [file gutjnl-2023-330748supp012.pdf]

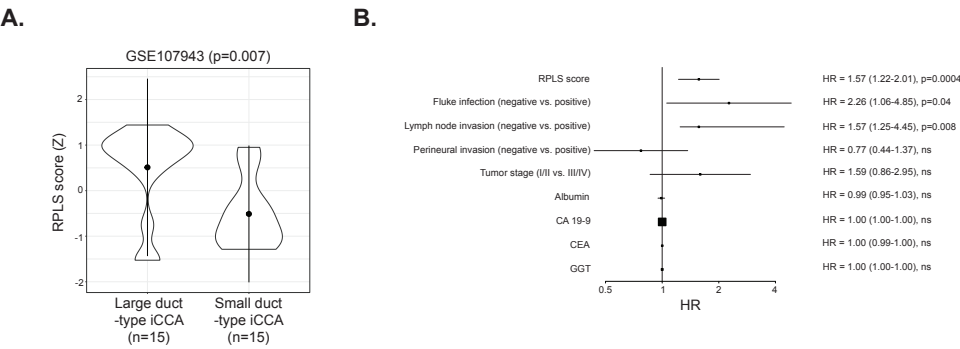

FIGURE S11

Supplement: Supplementary data [file gutjnl-2023-330748supp013.pdf]

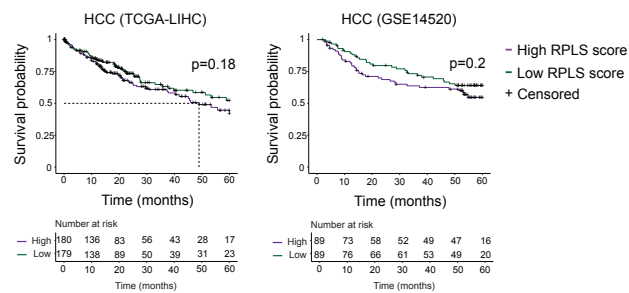

FIGURE S12

Supplement: Supplementary data [file gutjnl-2023-330748supp014.pdf]

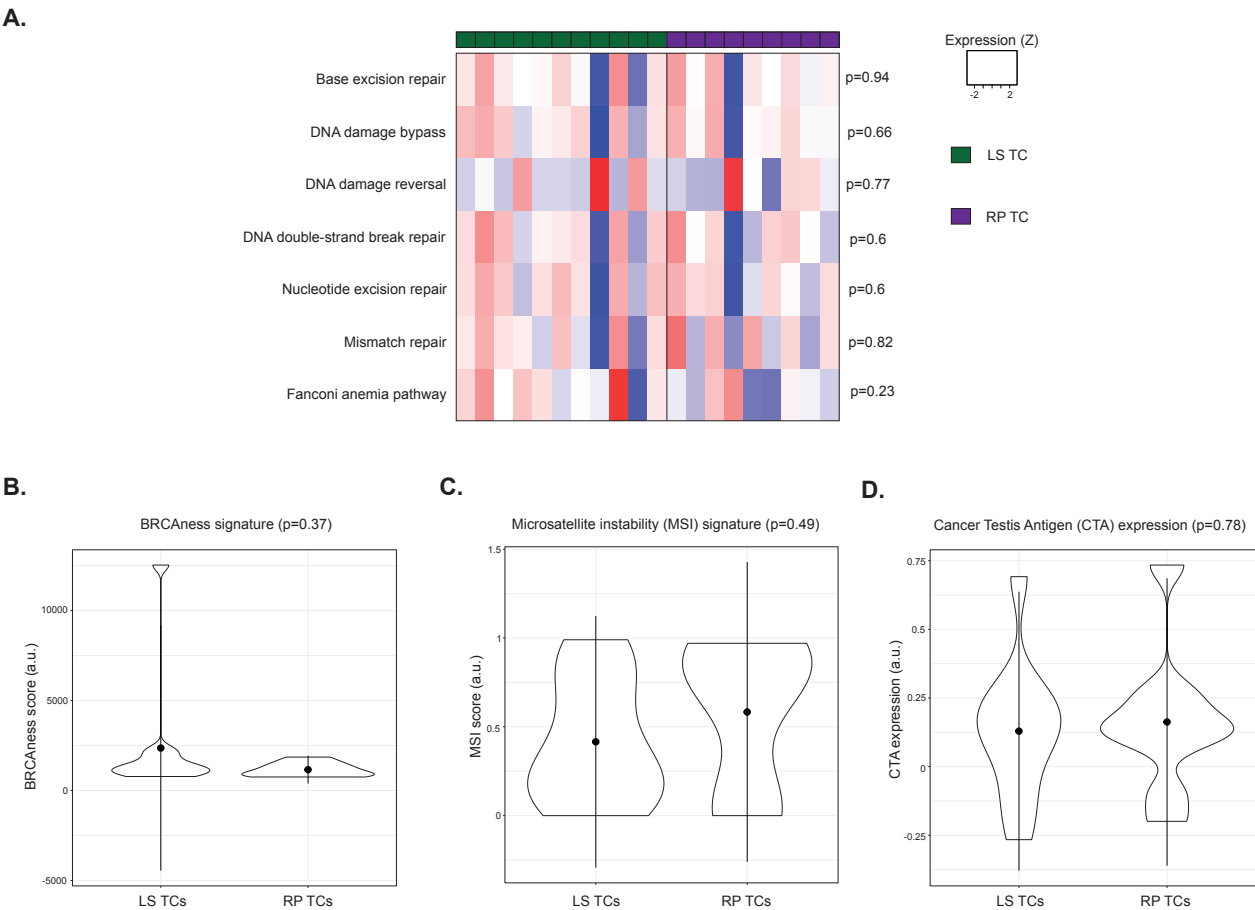

FIGURE S6

Supplement: Supplementary data [file gutjnl-2023-330748supp008.pdf]
